# GLT-1a glutamate transporter nanocluster localization is associated with astrocytic actin and neuronal Kv2 clusters at sites of neuron-astrocyte contact

**DOI:** 10.3389/fcell.2024.1334861

**Published:** 2024-02-01

**Authors:** Ashley N. Leek, Josiah A. Quinn, Diego Krapf, Michael M. Tamkun

**Affiliations:** ^1^ Department of Biomedical Sciences, Colorado State University, Fort Collins, CO, United States; ^2^ Molecular, Cellular and Integrative Neuroscience Program, Colorado State University, Fort Collins, CO, United States; ^3^ Department of Electrical and Computer Engineering, Colorado State University, Fort Collins, CO, United States; ^4^ Department of Biochemistry and Molecular Biology, Colorado State University, Fort Collins, CO, United States

**Keywords:** GLT-1, actin cytoskeleton, membrane diffusion, nanoclusters, Kv2.1 clusters, glutamate

## Abstract

**Introduction:** Astrocytic GLT-1 glutamate transporters ensure the fidelity of glutamic neurotransmission by spatially and temporally limiting glutamate signals. The ability to limit neuronal hyperactivity relies on the localization and diffusion of GLT-1 on the astrocytic surface, however, little is known about the underlying mechanisms. We show that two isoforms of GLT-1, GLT-1a and GLT-1b, form nanoclusters on the surface of transfected astrocytes and HEK-293 cells.

**Methods:** We used both fixed and live cell super-resolution imaging of fluorescent protein and epitope tagged proteins in co-cultures of rat astrocytes and neurons. Immunofluorescence techniques were also used. GLT1 diffusion was assessed via single particle tracking and fluorescence recovery after photobleach (FRAP).

**Results:** We found GLT-1a, but not GLT-1b, nanoclusters concentrated adjacent to actin filaments which was maintained after addition of glutamate. GLT-1a nanocluster concentration near actin filaments was prevented by expression of a cytosolic GLT-1a C-terminus, suggesting the C-terminus is involved in the localization adjacent to cortical actin. Using super-resolution imaging, we show that astrocytic GLT-1a and actin co-localize in net-like structures around neuronal Kv2.1 clusters at points of neuron/astrocyte contact.

**Conclusion:** Overall, these data describe a novel relationship between GLT-1a and cortical actin filaments, which localizes GLT-1a near neuronal structures responsive to ischemic insult.

## 1 Introduction

Glutamate is the most abundant excitatory neurotransmitter in the central nervous system, and therefore, it is imperative to maintain a low extracellular glutamate concentration to enhance the spatial and temporal resolution of glutamatergic signaling. To achieve efficient extracellular glutamate clearance against a steep electrochemical gradient, astrocytes couple glutamate uptake to the ionic movement of Na^+^, K^+^ and H^+^ using abundantly expressed glutamate transporters, such as GLT-1 and GLAST (Furuta et al., 1997). Excessive extracellular glutamate results in neuronal hyperactivity and subsequent neuronal death due to toxic levels of intracellular Ca^2+^ (Belov Kirdajova et al., 2020).

It is estimated that glutamate transporters represent 1% of total brain protein (Lehre and Danbolt, 1998) and that GLT-1 is responsible for approximately 90% of total glutamate uptake (Tanaka et al., 1997). GLT-1 is dense in the hippocampal astrocyte membrane, with an average of approximately 8,500 molecules/µm^2^ across the entire cell surface (Lehre and Danbolt, 1998), but with increased concentration in peri-synaptic astrocyte membranes (Chaudhry et al., 1995; Danbolt, 2001). One reason for this abundance may be related to their relatively slow transport cycle of 11–70 ms per glutamate molecule (Wadiche et al., 1995; Bergles and Jahr, 1998; Otis and Jahr, 1998), which is long compared to the lifetime of glutamate in the synaptic cleft (1.2 ms (Clements et al., 1992)). The high abundance of GLT-1 also limits glutamate spill over into other synaptic clefts, preventing excitation at inactive synapses (Asztely et al., 1997).

In addition to its localization, the mobility of active GLT-1 transporters in the membrane is important in shaping glutamate neurotransmission in the hippocampus (Murphy-Royal et al., 2015). Transporters without bound glutamate are relatively immobile, especially near synapses, but when glutamate is bound, transporter diffusion increases, thus allowing transporters to diffuse away from high concentrations of glutamate (Murphy-Royal et al., 2015; Al Awabdh et al., 2016). This mobility change is effectively responsible for replacing glutamate-bound transporters with unbound ones, conceivably to overcome slow transport. Given the importance of GLT-1 in regulating synaptic glutamate signals, it is vital to understand the mechanisms governing localization and diffusion of these transporters.

The majority of published work on GLT-1 localization has focused on localization adjacent to dendritic synapses (Chaudhry et al., 1995; Ventura and Harris, 1999; Danbolt, 2001; Minelli et al., 2001; Cholet et al., 2002; Genoud et al., 2006; Benediktsson et al., 2012; Murphy-Royal et al., 2015; Al Awabdh et al., 2016; Rose et al., 2017; Heller et al., 2020). However, a much smaller literature describes a functional and spatial relationship of astrocytic GLT-1 transporters and clusters of Kv2.1 channels on the neuronal soma (Misonou et al., 2008; Mulholland et al., 2008). Kv2.1 is a voltage-gated potassium channel highly expressed in central neurons (Trimmer, 1991). Under normal conditions, the majority of Kv2.1 channels are electrically silent and reside in μm-sized clusters on the neuronal surface (Lim et al., 2000; Misonou et al., 2005; O'Connell and Tamkun, 2005; Fox et al., 2013a) that represent sites of endoplasmic reticulum/plasma membrane (ER/PM) junctions (Du et al., 1998; Johnson et al., 2018; Kirmiz et al., 2018). In fact, Kv2 channels form ER/PM junctions via phosphorylation dependent binding to ER VAMP-associated proteins (VAPs). Interestingly, Kv2.1 clusters also serve as contacts between the neuronal soma and astrocyte or microglia processes (Du et al., 1998; Cserép et al., 2020). Kv2.1 clusters also act as indirect sensors of extrasynaptic glutamate, given that an extrasynaptic NMDA receptor-mediated Ca^2+^ current leads to calcineurin-mediated dephosphorylation of the Kv2.1 C-terminus, subsequent release of Kv2.1 channels from the ER, and thus declustering (Misonou et al., 2004; Misonou et al. 2005; Misonou et al. 2008; Fox et al., 2015). This declustering also results in a retraction of the ER from the neuronal plasma membrane (Fox et al., 2015) and is likely neuroprotective since peptides that block Kv2.1 binding to the ER reduce neuronal death in experimental stroke models (Schulien et al., 2020). Dephosphorylation of Kv2.1 channels also causes a location independent hyperpolarizing shift in the voltage-dependence of activation of the Kv2.1 channels that are conducting, such that the Kv2.1 potassium current activates earlier, preventing neuronal excitation in excitotoxic conditions (Misonou et al., 2004; Misonou et al., 2005). Interestingly, astrocytic GLT-1 has been reported to localize in net-like structures around Kv2.1 clusters in the somatosensory cortex (Misonou et al., 2008) although the mechanism of this localization is unknown.

GLT-1 contains eight transmembrane domains within almost 600 amino acids and can be expressed as one of three different splice variants, GLT-1a, GLT-1b and GLT-1c (Pines et al., 1992; Chen et al., 2002; Rauen et al., 2004). GLT-1a accounts for approximately 90% of the total GLT-1 population in the hippocampus, while GLT-1b makes up 6% with GLT-1c making up the rest (Holmseth et al., 2009). GLT-1a and GLT-1b differ only in the last 22 amino acids of the distal C-terminus (Holmseth et al., 2009) which gives GLT-1b the ability to bind PDZ domain proteins, such as PSD-95 (González-González et al., 2008). However, at this time, no function has been attributed to the unique amino acids in GLT-1a.

The aim of this study is to determine the factors governing GLT-1 localization and diffusion. We demonstrate extensive localization of GLT-1a, but not GLT-1b, nanoclusters at regions where actin filaments are in close contact with the astrocyte plasma membrane. Expression of a soluble GLT-1a C-terminus reduced localization associated with actin filaments, suggesting a GLT-1a specific C-terminus interaction is primarily responsible for the observed localization adjacent to cortical actin. Single-particle tracking of GLT-1a showed that diffusion was inhibited near cortical actin, an effect that was also eliminated by co-expression of the C-terminus. While glutamate enhanced overall GLT-1a diffusion, it did not alter the association of nanoclusters with actin, suggesting that glutamate primarily affects the motility of free transporters. Finally, we show that in hippocampal co-cultures of astrocytes and neurons astrocytic GLT-1 and actin colocalized in net-like structures around neuronal clusters of Kv2.1. Altogether, these data further elucidate mechanisms governing GLT-1 localization, particularly near insult-sensitive structures on the neuronal soma (Misonou et al., 2006; Misonou et al., 2008; Mulholland et al., 2008; Fox et al., 2015) that are involved in exocytosis (Feinshreiber et al., 2009; Feinshreiber et al., 2010; Deutsch et al., 2012; Du et al., 2018) and glia-neuron interaction (Du et al., 1998; Cserép et al., 2020).

## 2 Materials and methods

### 2.1 DNA constructs

For specific expression of proteins in astrocytes or neurons, the gfaABC1D and SYN promoters were used, respectively. GFP-GLT-1a-V5 and GFP-GLT-1b-V5 were generous gifts from Josef Kittler (University College London). The locations of these tags are illustrated in Supplementary Figure S1A. Ruby2-GLT-1a-V5 was made by digestion of mRuby2-C1 and GFP-GLT-1a-V5 with NheI and XhoI, with Ruby2 then ligated in place of GFP. The gfaABC1D promoter was inserted via PCR-mediated addition of restriction sites, AseI and NheI, to the ends of the gfaABC1D promoter from pAAV.gfaABC1D.GluSnFr.SV40 (a gift from Baljit Khakh, Addgene plasmid # 100889). gfaABC1D > GLT-1a-V5 was generated by restriction digest of gfaABC1D > Ruby2-GLT-1a-V5 with AgeI and BspEI to remove the Ruby2, followed by ligation. This construct was then sent to VectorBuilder (Chicago, IL), where it was cloned into an AAV vector and packaged it into an AAV5 virus. GFP-Actin was obtained from Takara Bio (Mountain View, CA). gfaABC1D > Ruby2-Actin was generated by restriction digest of gfaABC1D > Ruby2-GLT-1a-V5 and GFP-Actin with NheI and XhoI and subsequent ligation of the NheI-GFP-Actin-XhoI into the XhoI-gfaABC1D-NheI vector. AAV9:SYN > AMIGO-GFP was used to label the endogenous Kv2.1 clusters in neurons without increasing Kv2.1 expression. This virus was also packaged by VectorBuilder. To create the gfaABC1D > Ruby2-GLT-1a-CT construct, expressing only the GLT-1a C-terminal 81 amino acids, PCR was used to generate a fragment flanked by BspEI and BamHI restriction sites (Primers: 5′ GCT​TAC​TCC​GGA​TAT​CAC​CTT​TCC​AAG​TCC 3′ and 5′ AGT​CCG​GGA​TCC​TTA​TTT​TTC​ACG​TTT​CCA​AGG 3’). This fragment was then digested with BspEI and BamHI and ligated into the gfaABC1D > Ruby2-GLT-1a-V5 cut with the same enzymes to create a Ruby2-tagged GLT-1a C-terminus.

### 2.2 Cell culture, transfection and labeling

Hippocampal co-cultures of neurons and astrocytes were isolated from E18 rat brains. Pregnant rats were deeply anaesthetized with isoflurane, as outlined in a protocol approved by the Institutional Animal Care and Use Committee of Colorado State University (protocol ID: 15–6130A). Embryos of both sexes were used to generate cultures, and thus the cells are a mixed population of male and female origins. Hippocampal cells were dissociated and cultured as previously described for neurons (Bartlett and Banker, 1984a; Bartlett and Banker, 1984b; Brewer et al., 1993). Cultures were plated on glass-bottom 35 mm dishes with No. 1.5 coverslips (MatTek, Ashland, MA) coated with poly-L lysine (Sigma-Aldrich, St. Louis, MO) in borate buffer, and plated in a plating medium composed of 5% FBS, Neurobasal (Gibco/Thermo Fisher Scientific, Waltham, MA), B27 Plus Supplement (Gibco/Thermo Fisher Scientific, Waltham, MA), Penicillin/Streptomycin (Cellgro/Mediatech, Manassas, VA), and GlutaMAX (Gibco/Thermo Fisher Scientific). After 24 h in plating media, the media was replaced with maintenance medium which was identical to the plating medium without FBS. Co-cultures were maintained at 37°C under 5% CO_2_.

At DIV7, cultures were transfected using DNA, Lipofectamine 2000 (Invitrogen, Life Technologies, Grand Island, NY), and OptiMEM for experiments the following day. The following amounts of DNA were transfected per dish: gfaABC1D > Ruby2-GLT-1a-V5 (0.5 µg), gfaABC1D > GLT-1a-V5 (0.5 µg), gfaABC1D > GLT-1b-V5 (0.5 µg), gfaABC1D > Ruby2-GLT-1aCT (1 µg), GFP-Actin (0.2 µg). At DIV7, rat hippocampal co-cultures were infected with 1 × 10^10^ genocopies of AAV9:SYN-AMIGO-GFP and 5 × 10^9^ genocopies of AAV5:gfaABC1D-GLT-1a-V5 for single particle tracking experiments in co-culture.

For DIV8 experiments, after 24 h the cultures were transferred to imaging saline (126 mM NaCl, 4.7 mM KCl, 2.5 mM CaCl_2_, 0.6 mM MgSO_4_, 0.15 mM NaH_2_PO_4_, 0.1 mM ascorbic acid, 8 mM glucose, and 20 mM HEPES, pH 7.4, 300 mOsm) containing 1:1000 αV5-CF640 (Biotium, Hayward, CA) for 3 min at 37°C. The cultures were washed 2 times with imaging saline and then transferred to the TIRF microscope (described below) for experimentation. For experiments where neurons and astrocytes were imaged simultaneously, cells were cultured for 14 days and subsequently, either transferred to imaging saline (described above) or prepared for immunocytochemistry, described below.

HEK-293 cells were maintained in 10 cm dishes (CellTreat #229620, Pepperell, MA) at 37°C under 5% CO_2_ in DMEM (Corning #10–013-CV, Corning, NY) supplemented with 10% FBS. For transfections, cells were trypsinized and electroporated (BioRad GenePulse Xcell, Berkeley, CA) with 1 mg Ruby2-GLT-1a-V5 or Ruby2-GLT-1b-V5. Following transfection, cells were plated on Matrigel (Corning, Corning, NY) coated glass-bottom 35 mm dishes with No.1.5 coverslips (MatTek, Ashland, MA) and imaged the following day.

### 2.3 Microscopy

Total internal reflection fluorescence (TIRF) microscopy was performed on a Nikon Eclipse Ti fluorescence microscope. Images were acquired with an Andor iXon (DU-897) camera and 100X Plan Apo TIRF, NA 1.49 objective lens. Diode lasers (405, 488, 561, 640 nm, 100 mW) were controlled with an acousto-optic tunable filter (AOTF) and excitation occurred with lasers at an incident angle of 63°, allowing the evanescent wave to penetrate approximately 144 nm at a wavelength of 488 nm. Emitted light was collected through the proper bandpass filters. Videos were acquired at 20 Hz (50 ms exposure) for 2000 total frames with a beam splitter, such that emitted green and far-red could be imaged simultaneously. All imaging was performed at 37°C using a heated stage and objective heater.

Spinning disk confocal microscopy was performed on a Yokogawa (Musashino, JP) based CSUX1 system with an Olympus (Tokyo, JP) IX83 inverted stand, and coupled to an Andor (Abingdon, GB) laser launch containing 405, 488, 568, and 637 nm diode lasers, 100–150 mW each. Images were collected using two Andor iXon EMCCD cameras (DU-897), oriented perpendicularly, and a 100X Plan Apo, 1.4 NA objective. To split the emitted fluorescence when imaging concurrently for single particle tracking and fluorescence recovery after photobleaching, a dichroic mirror was used. This system is equipped with the ZDC constant focus system and a Tokai Hit chamber and objective heater. Images were collected using MetaMorph software (version 7.8.13.0).

### 2.4 Photobleaching steps to determine number of transporters per nanocluster

HEK-293 cells or DIV8 primary astrocytes expressing Ruby2-GLT-1a-V5 or Ruby2-GLT-1b-V5 were labeled with a rabbit antibody directed against the V5 epitope conjugated with CF640 (anti-V5-CF640, Biotium), fixed using 4% paraformaldehyde and then washed using 1X Phosphate Buffered Saline. The fixed cells were then imaged on the TIRF microscope for 30,000 frames at 20 Hz and 30% power of the 100 mW 640 nm laser. Using ImageJ, local maxima were identified in the first frame of the movie and small circular ROIs were drawn around each point. The ROIs were then used to measure the fluorescence of that spot over the entire course of the movie. Using a moving average of 50 frames, the smallest sustained drops in fluorescence, which indicate a single bleached molecule, were used to determine the number of fluorescent molecules in the initial nanocluster. According to specifications from Biotium, each anti-V5 antibody was conjugated with five CF640 molecules. Using this knowledge, we estimated the total number of antibodies bound per nanocluster. Due to the difficulty in assessing antibody binding efficiency, we did not convert number of antibodies to number of transporters in each nanocluster.

### 2.5 Super-resolution radial fluctuations (SRRF)

**FIGURE 1:**
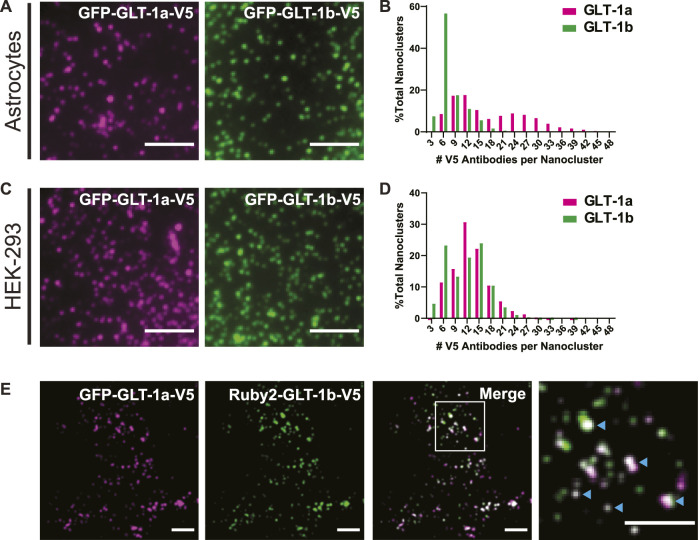
GLT-1 forms nanoclusters on the surface of both astrocytes and HEK-293 cells. **(A)** Representative images of nanoclusters of transfected GLT-1a and GLT-1b in fixed DIV8 hippocampal astrocytes as imaged with TIRF microscopy. **(B)** Distribution of GLT-1a (magenta) or GLT-1b (green) antibodies per nanocluster in astrocytes. Plot shows anti-V5 antibodies bound per nanocluster. GLT-1a showed a bimodal distribution, with peaks at 9–12 antibodies and 24–27 antibodies per nanocluster, while GLT-1b favored smaller nanoclusters of 6 antibodies. **(C)** Representative images of nanoclusters of GLT-1a (left, magenta) and GLT-1b (right, green) in HEK-293 cells. **(D)** Distribution of GLT-1a (magenta) or GLT-1b (green) antibodies per nanocluster in HEK-293 cells. GLT-1a favored nanoclusters of 12 antibodies, while GLT-1b showed a bimodal distribution with peaks at both 6 and 15 antibodies per nanocluster. **(E)** Astrocytes expressing GFP-GLT-1a-V5 and Ruby2-GLT-1b-V5 were labeled with a V5 antibody. The signal from the V5 was used to determine the fluorescence contribution of GLT-1a and GLT-1b to surface nanoclusters. These astrocytes displayed surface nanoclusters which contained both GLT-1 isoforms (cyan carets in the enlarged panel, far right). Scale bars represent 5 µm in all panels.

To overcome limitations in lateral resolution due to Abbe’s diffraction limit, we applied the super-resolution radial fluctuations (SRRF) approach to achieve better than 100 nm lateral resolution (Gustafsson et al., 2016) over a shorter time frame, with various emitting fluorophore densities. SRRF was used for Figure 1, Figures 3, 5, and Supplementary Figure S5, with the NanoJ-SRRF plugin for ImageJ (Laine et al., 2019)

For images acquired on the TIRF microscope, 2000 continuous frames were acquired at 20 Hz on live culture samples. These videos were obtained through a beam splitter, which allowed the simultaneous collection of emissions from two different fluorophores. Subsequently, these videos were background subtracted and processed with SRRF. The SRRF algorithm was applied to non-overlapping sets of 50 frames, and therefore final super-resolution frame rates were 1 frame every 2.5 s.

For images acquired on the spinning disk confocal microscope, 200 frames for each wavelength were acquired sequentially at each focal plane in fixed co-culture samples. Subsequently, these videos were background subtracted and processed with SRRF. The SRRF algorithm was applied over 200 frames, and therefore one super-resolution image represented each focal plane.

### 2.6 Single particle tracking

DIV8 rat hippocampal co-cultures expressing GLT-1a-V5 or GLT-1b-V5 were labeled with 1:1000 αV5-CF640 for 3 min at 37°C, as described above. Astrocytes co-expressing fluorescent organelle markers and GLT-1a, GLT-1b, and/or GLT-1aCT were identified, and single frames were imaged. The beam splitter was then inserted, and 2000-frame movies were acquired before and after chemical intervention (100 µM glutamate). 1 mL of 200 µM glutamic acid in imaging saline was applied to the dish with 1 mL normal imaging saline while on the microscope stage and allowed to bind to GLT-1 for 3 min before acquiring “+Glu” movies.

For processing of TIRF movies, individual channels were aligned using DIC images captured through the beam splitter. Due to the widespread coverage of astrocytes across the glass surface of the dish, multi-colored beads, conventionally used for aligning beam splitter images, could not be used. DIC images acquired through the beam splitter were aligned using the AutoAlign plugin in ImageJ. These alignment settings were then applied to the other channels.

DIV14 rat hippocampal co-cultures infected with AAV9:SYN-AMIGO-GFP and AAV5:gfaABC1D-GLT-1a-V5 were labeled with αV5-CF640 for 3 min in imaging saline, and subsequently transferred to the spinning disk confocal microscope for imaging. The chamber was kept at 37°C for the entirety of imaging. Using a dual-camera system and a dichroic mirror, AMIGO and GLT-1a were imaged simultaneously at 20 Hz for 2000 frames. Before imaging, a dish covered in TetraSpeck beads (Invitrogen) was imaged through both cameras for alignment purposes.

Images containing GLT-1 molecules were background subtracted and a Gaussian filter with a standard deviation of 0.7 pixels was applied to each frame in ImageJ. The channel images containing actin or AMIGO underwent processing for SRRF analysis, such that 2000 frames were temporally correlated and averaged to 40 frames (Gustafsson et al., 2016). This sequence was converted to binary images that were eventually used to identify nanoclusters. Individual GLT-1 molecules were tracked using the U-track algorithm in MATLAB (Jaqaman et al., 2008), as previously described (Weigel et al., 2013; Akin et al., 2016). Subsequently, tracks were segmented and classified based on the spatial relationship to actin using a custom MATLAB code into ‘on’ states, when they were found to colocalize with actin, and ‘off’ states otherwise. A 3-pixel region between the two regions was excluded, so that trajectories were identified within each region with a high degree of certainty. Trajectory segments in each state (on or off) were discarded if they did not remain for at least 40 consecutive frames (2 s) in the same state. The trajectories in each region were then used to calculate individual time-averaged mean square displacements (MSD)

$$\bar{\delta^{2}\left(\Delta t\right)}=\frac{1}{T-\Delta t}\int_{0}^{T-\Delta t}{\left|r\left(t+\Delta t\right)-r\left(t\right)\right|}^{2}dt,$$ (1)

where **r**(*t*) is the two-dimensional particle position at time *t*, and Δ*t* is the lag time. The individual MSDs of all molecules (Eq. 1) were then averaged using a custom MATLAB code. When the MSD exhibited a linear behavior in lag time, the diffusion coefficient *D* was calculated using the equation

$$\text{MSD}\left(∆t\right)=4D\left(∆t\right)+{4\sigma }^{2}\;.$$ (2)

where the term 4s^2^ is due to tracking localization errors. When the MSD exhibited non-linear behavior, i.e., Eq. 2 does not hold and the tracers displayed anomalous diffusion, the generalized diffusion coefficient K and anomalous exponent *α* were calculated using the equation

$$\text{MSD}\left(∆t\right)={\;K∆t}^{\alpha \;}+{4\sigma }^{2}.$$ (3)

The coefficient *K* represents the area explored by a molecule in a unit time and the exponent *α* describes the deviations from Brownian motion. These deviations may be due to crowding, transient confinement, or dynamic interactions (Weigel et al., 2011; Krapf, 2015). When *α* = 1, Brownian motion is recovered (i.e., a linear MSD), while α≠1 indicates anomalous diffusion with 1 < *α* < 2 being a superdiffusive process, and 0 < *α* < 1 a subdiffusive process, which is commonly observed in biological samples (Metzler et al., 2014; Krapf, 2015).

### 2.7 Nanocluster measurements

Nanoclusters are smaller than the diffraction limit, and thus the location of stable nanoclusters was determined by identifying the center of a Gaussian fit of point-spread functions from 50 averaged frames of GLT-1 movies. As a control, an equal number of XY coordinates were randomly generated in MATLAB to form a random pixel image (Fox et al., 2013b). These XY coordinates were then compared to 50 averaged frames of actin imaging and determined to be “on” or “off” actin. For calculating distance to actin, the 50 averaged frames of actin imaging were converted into an Euclidean distance map (EDM) in MATLAB (Fox et al., 2013a). Nanocluster centers were used to obtain their distance to the nearest actin filament using the EDM.

### 2.8 Immunofluorescence

Cells were fixed with 4% paraformaldehyde in imaging saline (described above) for 10 minutes, followed by permeabilization and block for 15 min at room temperature in 10% goat serum with 0.2% Triton-X 100. Cultures were incubated overnight at 4°C in primary antibodies at the following dilutions: 1:400 mouse anti-Kv2.1 (NeuroMab, Davis, CA) and 1:1000 rabbit anti-EAAT2 (Abcam, Cambridge, MA). The anti-EAAT antibody was made against the C-terminal 24 amino acids of GLT1a. Cells were washed and then incubated in secondary antibody for 1 h at room temperature in 10% goat serum with 1:1000 goat anti-mouse Alexa-Fluor 647 and 1:1000 goat anti-rabbit Alexa-Fluor 488 (Invitrogen, Carlsbad, CA). Cultures were then washed and mounted with Aqua Poly/Mount (Polysciences, Warrington, PA) and subsequently imaged with the spinning disk confocal microscope.

### 2.9 Image processing and analysis

Image processing was done in Volocity (v 6.2) and ImageJ (v. 1.52). Analysis was completed in MATLAB (R2019a) and Origin (v 2023b). Statistics and graphs were generated in GraphPad Prism 9. When indicted, means are presented ± the standard error of the mean (SEM). In cases where multiple groups were compared, one-way ANOVAs were used followed by *post hoc* Sidak’s tests to compare specific groups to one another, unless otherwise noted.

## 3 Results

### 3.1 GLT-1 forms nanoclusters on the surface of astrocytes and HEK-293 cells

Previous work using freeze-fracture electron microscopy suggested that GLT-1 forms small clusters of approximately 200 nm in diameter when exogenously expressed in baby hamster kidney (BHK) cells (Raunser et al., 2006). To determine whether we could observe surface-localized nanoclusters using fluorescence microscopy, we expressed GFP-GLT-1a-V5 and GFP-GLT-1b-V5 in primary astrocytes and HEK-293 cells and labeled the extracellular V5 epitope with the anti-V5 antibody conjugated to CF640. This antibody labeling was performed after fixation to ensure that nanoclustering was not enhanced by the bivalent nature of the antibody combined with multiple V5 epitopes. As illustrated by TIRF imaging, both isoforms, GLT-1a (magenta) and GLT-1b (green), formed nanoclusters in astrocytes (Figure 1A) and HEK-293 cells (Figure 1C). Note that even in unfixed live cells the V5 antibody did not alter the appearance of GFP-GLT-1 on the surface (Supplementary Figure S2, compare panels C and D). Transfected GFP-GLT-1a and GFP-GLT-1b trafficked well, localizing predominantly to the astrocyte surface (Supplementary Figures S2A, B). Thus, the inserted V5 and GFP tags do not alter ER exit and delivery to the cell surface. The expression level of transfected GLT-1a was on average 2.2-fold greater that the endogenous transporter (*p* = 0.0016, N = 5 cell pairs) as determined by antibody labeling of transfected and untransfected astrocytes.

To estimate the number of transporters per nanocluster, we labeled the V5 epitope with an antibody conjugated to a known number of CF640 molecules, and subsequently photobleached the entire cell surface, such that we could resolve bleaching events of single CF640 fluorophores (similar to the approaches used previously (Ulbrich and Isacoff, 2007; Fox et al., 2013b)). Using the magnitude of a single bleaching event and the number of CF640 molecules per antibody, the original number of antibodies bound to a nanocluster was estimated. A histogram detailing the number of antibodies per nanocluster reveals GLT-1a nanoclusters showed a bimodal distribution, with peaks at 9–12 antibodies and 24–27 antibodies per nanocluster in astrocytes (Figure 1B, magenta bars). In contrast, most GLT-1b nanoclusters usually contained only 6 antibodies (Figure 1B, green bars). However, these numbers are likely an underestimation of the true density of GLT-1 nanoclusters in astrocytes due to high endogenous expression of GLT-1. In HEK-293 cells, which lack endogenous GLT-1 expression, we observed a peak for GLT-1a at 12 antibodies per nanocluster (Figure 1D, magenta bars). In contrast, GLT-1b showed a bimodal distribution, with peaks at both 6 antibodies and 15 antibodies per nanocluster (Figure 1D, green bars). Notably, the population of GLT-1a nanoclusters with more transporters was absent in the HEK-293 data set, perhaps suggesting astrocyte-specific expression of a GLT-1a binding partner.

While GLT-1a and GLT-1b subunits have been shown to co-localize in astrocytes (González-González et al., 2009), these past studies did not distinguish between GLT-1 transporters localized to the surface or in internal stores. To determine whether GLT-1a and GLT-1b could occupy the same nanoclusters on the surface of astrocytes, GFP-GLT-1a-V5 and Ruby2-GLT-1b-V5 were co-expressed and labeled with the CF640-conjugated V5 antibody described above. Using the V5 signal, which represents transporters on the surface, we determined that GFP-GLT-1a-V5 and Ruby2-GLT-1b-V5 signals often resided in the same surface nanoclusters, as evidenced by the white co-localization signal in the merged image and cyan carets (Figure 1E). When examining nanoclusters for expression of both isoforms in DIV7 astrocytes we found that 98.3% of Ruby-GLT-1a clusters (906 nanoclusters) contained some degree of GFP-GLT-1bV5 and 98.6% of GFP-GLT-1bV5 clusters (971 nanoclusters) contained some Ruby-GLT-1aV5 (5 cells examined). This degree of colocalization is not surprising given that GLT1 assembly occurs in the ER and transfected transporters most likely assemble with themselves as opposed to the endogenous transporters already at steady state expression (Kalandadze et al., 2004). The ratio of GLT-1a to GLT-1b in each nanocluster ranged widely as illustrated in Figure 1E, ranging from being equal to a 10-fold difference, likely reflecting varying efficiency in the ER-based isoform assembly.

### 3.2 Astrocytic GLT-1a nanoclusters align with cortical actin filaments

Membrane protein clustering is driven by several factors, including lipid-protein interactions (Harding and Hancock, 2008; Nussinov et al., 2015), protein-protein interactions between a membrane protein and organelle protein (Johnson et al., 2018; Kirmiz et al., 2018), and cytoskeletal corralling (Garcia-Parajo et al., 2014; Krapf, 2015; Kalappurakkal et al., 2020; van Deventer et al., 2021). Previous studies have used pharmacology and biochemical approaches to propose the cytoskeleton is involved in regulation of glutamate transporter localization (Marie et al., 2002; Zhou and Sutherland, 2004; Piniella et al., 2018). However, none of these studies have shown co-localization of GLT-1 transporters with the actin cytoskeleton, although some have noted GLT-1 localization in actin-rich filopodia (Zhou and Sutherland, 2004; Benediktsson et al., 2012; Hayashi and Yasui, 2015). To determine whether the GLT-1 nanoclusters shown in Figure 1 were associated with actin structures in close contact with the plasma membrane, hippocampal astrocytes were transfected with Ruby2-GLT-1a-V5 or Ruby2-GLT-1b-V5 and GFP-Actin, and subsequently labeled with a V5 antibody conjugated to CF640. Using a combination of super-resolution radial fluctuations (SRRF) analysis and TIRF microscopy, we correlated the location of GLT-1 nanoclusters with actin that was within 150 nm of the plasma membrane. By using SRRF, we achieved better than 100 nm lateral resolution, which ensured more precise localization of both nanoclusters and cortical actin filaments.

**FIGURE 2:**
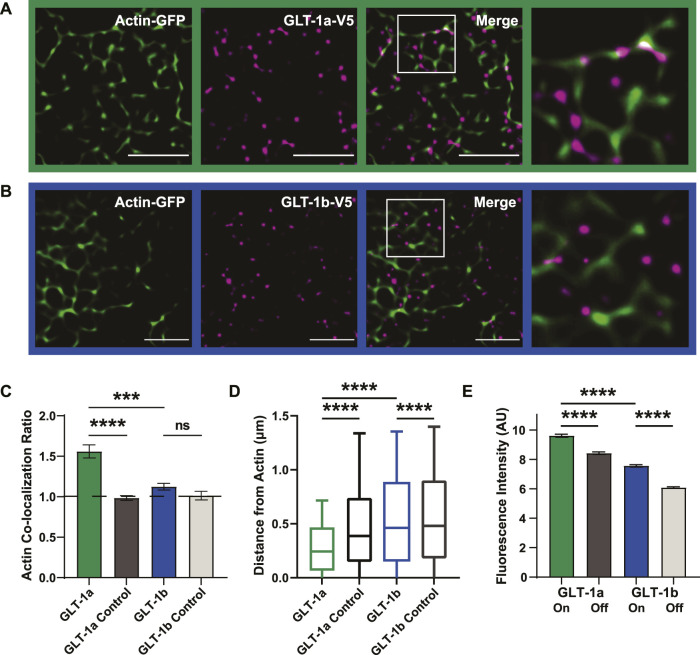
GLT-1 transporters co-localize with actin near the surface of the plasma membrane. DIV8 astrocytes co-expressing GFP-actin and Ruby2-GLT-1a-V5 were labeled with a V5 antibody conjugated to CF640, imaged using TIRF microscopy and subsequently, processed using SRRF to improve spatial resolution. **(A)** GLT-1a nanoclusters (magenta) co-localize with cortical actin filaments (green) in primary astrocyte cultures. **(B)** GLT-1b nanoclusters (magenta) rarely co-localize with cortical actin filaments (green) in primary astrocytes. **(C)** The ratio of the fraction of nanoclusters co-localized within actin to the fraction the cell surface covered by actin was measured for each cell, such that a ratio of 1 would indicate random nanocluster distribution, above 1 enhanced co-localization, and below 1 avoidance of actin. These ratios reveal a concentration of GLT-1a molecules on actin filaments (green bar, N = 58 cells, h_col_ = 1.56 ± 0.08), compared to a matched random pixel control (dark grey bar, N = 58 cells, h_col_ = 0.98 ± 0.03, ANOVA F = 13.65, *p* < 0.0001). GLT-1b (N = 17 cells, h_col_ = 1.12 ± 0.04) was not significantly concentrated on actin compared to a random pixel control (N = 17 cells, h_col_ = 1.02 ± 0.05, *p* = 0.998). GLT-1a nanoclusters were also significantly concentrated on actin compared to GLT-1b (*p* < 0.001). **(D)** Distance of both GLT-1a (left) and GLT-1b (right) nanoclusters from actin filaments. GLT-1a nanoclusters (median = 0.31 µm, N = 622,826 nanoclusters) were localized significantly closer to actin filaments than the random pixel control (median = 0.50 µm, N = 622,826 nanoclusters, *p* < 0.0001). GLT-1b nanoclusters (median = 0.54 µm, N = 158,864 nanoclusters) were localized slightly closer to actin than the random pixel control (median = 0.56 µm, N = 158,864 nanoclusters, *p* < 0.0001). Notably, GLT-1a nanoclusters were localized significantly closer to actin filaments compared to GLT-1b nanoclusters (*p* < 0.0001). Box plots represent the median and interquartile range. Bars represent the 10th—90th percentile. **(E)** GLT-1a nanoclusters had significantly greater fluorescence intensity on actin (7.66 ± 0.03 arbitrary units (AU), 57 cells, N = 34,774 nanoclusters) than off actin (6.05 ± 0.01AU, 57 cells, N = 219,464 nanoclusters, *p* < 0.0001). GLT-1b nanoclusters were also significantly different in fluorescence intensity on actin (5.81 ± 0.03, 17 cells, N = 13,365 nanoclusters) *versus* off actin (4.40 ± 0.01 AU, 17 cells, N = 99,045 nanoclusters, *p* < 0.0001). In addition, GLT-1a nanoclusters also had significantly larger fluorescence intensity than GLT-1b nanoclusters (*p* < 0.0001). Scale bars are 5 µm.

GLT-1a nanoclusters appeared to be often localized adjacent to the actin cytoskeleton (Figure 2A), shown by the magenta nanoclusters often co-localizing with, or directly adjacent to, actin filaments (green). In contrast, GLT-1b nanoclusters were much less likely to associate with actin filaments (Figure 2B). To quantitatively assess nanocluster association with cortical actin filaments, the colocalization ratio h_col_ was obtained by dividing the ratio of the fraction of GLT-1 nanoclusters localizing to actin regions by the cell surface area fraction covered by actin as illustrated in Figure 2C. In this characterization, a colocalization ratio of one indicates that nanoclusters are randomly distributed, while ratios greater-than or less-than one indicate concentration or exclusion from actin, respectively. GLT-1a nanoclusters were significantly concentrated near actin filaments (h_col_ = 1.56 ± 0.08), compared to a matched random pixel control (h_col_ = 0.98 ± 0.03, *p* < 0.0001, Figure 2C). GLT-1b (h_col_ = 1.12 ± 0.04) was not significantly concentrated on actin compared to a random pixel control (h_col_ = 1.02 ± 0.05, *p* = 0.998, Figure 2C). These data suggest that GLT-1a nanoclusters are specifically concentrated along or near actin filaments, perhaps due to the amino acids in the distal C-terminus, which are different in GLT-1a and GLT-1b.

To further assess the spatial relationship between each GLT-1 nanocluster and actin, we measured the distance of each nanocluster to the nearest actin filament. GLT-1a nanoclusters were localized close to actin, with a median distance of 0.31 µm, compared to 0.50 µm for randomly generated pixels (Figure 2D, *p* < 0.0001). GLT-1b was also localized slightly closer to actin (median = 0.54 µm) than the random pixel control (median = 0.56 µm, *p* < 0.0001, Figure 2D).

To test whether the cluster size depends upon their association with actin filaments, we measured the fluorescence intensity of V5 antibody-labeled GLT-1 nanoclusters on actin filaments *versus* those off actin filaments. The fluorescence intensity in each nanocluster should scale with the number of transporters within the nanocluster, as was the case in Figure 1. GLT-1a nanoclusters had significantly higher fluorescence intensity on actin than off actin (Figure 2E, *p* < 0.0001), and GLT-1b nanoclusters had significantly lower fluorescence intensity than GLT-1a nanoclusters (*p* < 0.0001). Although GLT-1b nanoclusters were not concentrated near actin filaments, those nanoclusters that did co-localize with actin also had significantly higher fluorescence intensity than those off actin (*p* < 0.0001). These data suggest the two populations of GLT-1a nanoclusters identified in Figure 1B represent nanoclusters localized to different compartments of the plasma membrane, with the cortical actin environment associating with a greater number of transporters per nanocluster. Altogether, these data support a specific preference of the GLT-1a isoform for localization near actin filaments. In addition, we examined the stability of GLT-1a nanoclusters within 300 nm of astrocytic cortical actin. As illustrated in Supplementary Figure S3, some nanoclusters were very stable with respect to location and intensity over 12 s of imaging while others were very dynamic, i.e., appearing only transiently.

### 3.3 GLT-1a-actin association is disrupted by cytosolic GLT-1a C-terminus expression

**FIGURE 3:**
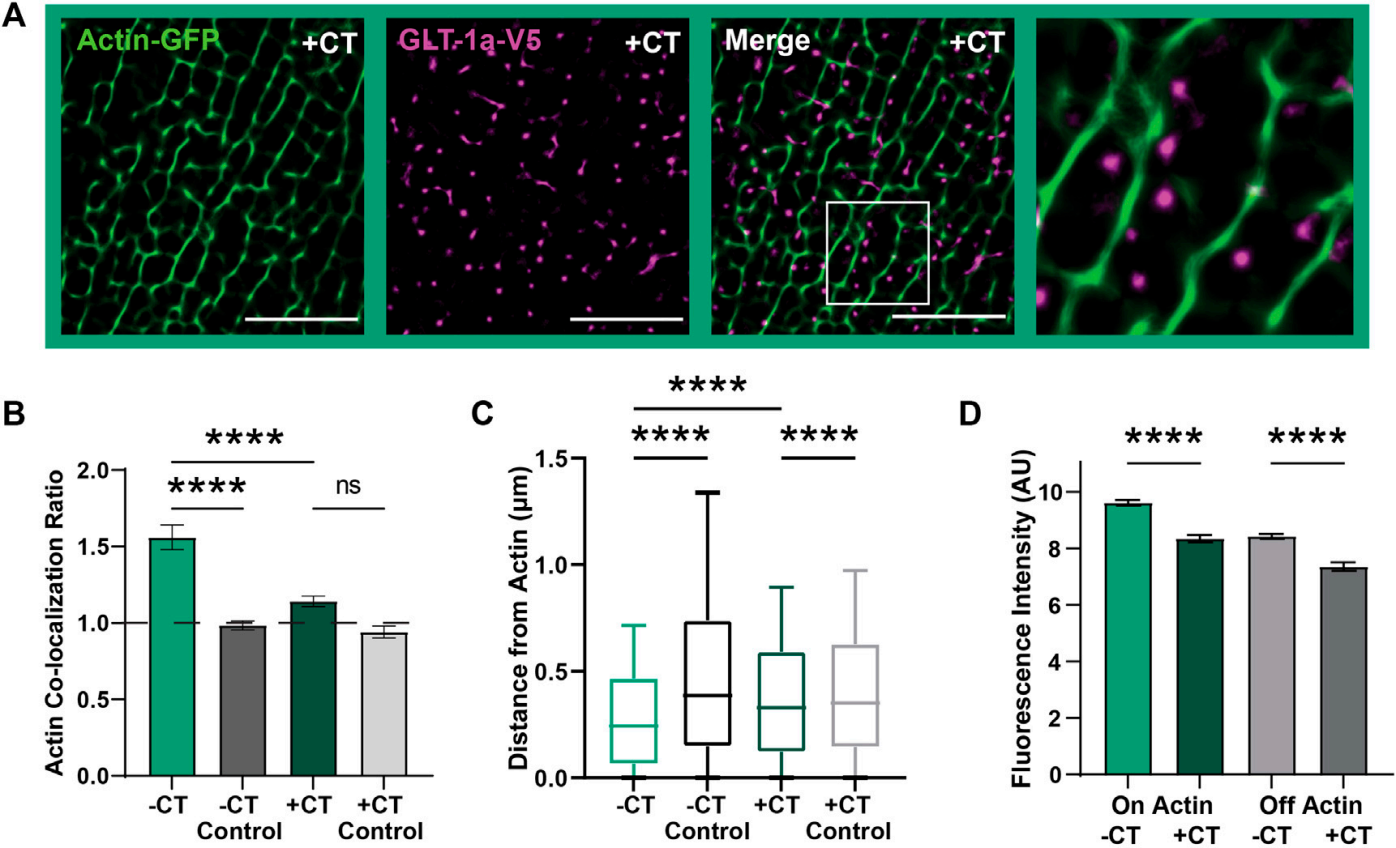
GLT-1a-Actin interaction disrupted by cytosolic GLT-1a C-terminus expression. DIV8 astrocytes co-expressing GFP-actin, GLT-1a-V5, and Ruby2-GLT-1a-CT were labeled with a V5 antibody conjugated to CF640, imaged using TIRF microscopy and subsequently, processed using SRRF to improve spatial resolution. **(A)** Representative images of GLT-1a-V5 nanoclusters (magenta) overlying Actin-GFP filaments (green) with a co-expressed cytosolic GLT-1a C-terminus (+CT). **(B)** A measure of concentration on actin shows a significant decrease with co-expression of a competing GLT-1a C-terminus (h_col_ = 1.14 ± 0.03, N = 28 cells, *p* < 0.0001). GLT-1a + CT was not significantly concentrated on actin relative to a random pixel control (h_col_ = 0.94 ± 0.04, *p* = 0.38). **(C)** GLT-1a nanoclusters (median = 0.31, N = 622,826 nanoclusters) were localized further from actin filaments when co-expressed with the CT (median = 0.37, N = 315,362 nanoclusters, *p* < 0.0001). Box plots represent the median and interquartile range. Bars represent the 10th—90th percentile. **(D)** Co-expression of the cytosolic GLT-1a CT significantly decreased nanocluster fluorescence intensity on actin (mean ± SEM = 5.71 ± 0.003 AU, 28 cells, N = 26,307 nanoclusters, *p* < 0.0001) and off actin (mean ± SEM = 4.71 ± 0.008 AU, 28 cells, N = 193,666 nanoclusters, *p* < 0.0001). Scale bars are 5 µm.

The observed relationship between actin and GLT-1 nanoclusters appears to be specific to the GLT-1a isoform. GLT-1a and GLT-1b differ only in the distal amino acids of the C-terminus as illustrated in Supplementary Figure S1B, with GLT-1a having an additional 21 unique amino acids which may have a specific binding partner. Therefore, we determined whether expression of the cytosolic GLT-1a C-terminus interferes with GLT-1a nanocluster localization relative to actin. A Ruby2-tagged GLT-1a C-terminus (CT) was expressed in astrocytes, and the association of GLT-1a nanoclusters with actin was assessed as described above. When co-expressed with the CT, GLT-1a nanoclusters (magenta) co-localized less often with actin filaments (green) compared to cells without the CT expressed (Figures 3A, B, *p* < 0.0001), suggesting that the distal C-terminus of GLT-1a is vital in localizing nanoclusters near actin filaments. In addition, distribution analysis showed that GLT-1a nanocluster distance to actin filaments significantly increased with co-expression of the CT (median = 0.37 µm, *p* < 0.0001, Figure 3C).

Furthermore, GLT-1a nanocluster fluorescence intensity near actin filaments was also significantly decreased by co-expression of the CT (Figure 3D, *p* < 0.0001). Interestingly, nanocluster fluorescence intensity off actin was also decreased by expression of the CT (*p* < 0.0001), suggesting that the C-terminus is important in regulating the number of transporters per nanocluster, regardless of actin localization. Together these data suggest that the GLT-1a C-terminus is important in localizing nanoclusters near actin filaments and regulating the number of transporters per nanocluster. Given the association between GLT-1a and actin we postulated that actin depolymerization would alter nanocluster intensity or cell surface density. However, as shown in Supplementary Figure S4, 5 h of Swinholide A treatment depolymerized astrocytic actin without altering the GLT-1a nanoclusters themselves. Perhaps an unknown actin-associated interactor mediates the relationship between cortical actin and GLT-1a and is sufficient to maintain nanoclusters that have already formed even after F-actin removal.

### 3.4 GLT-1a diffusion is influenced by actin and the cytosolic GLT-1a C-terminus

**FIGURE 4:**
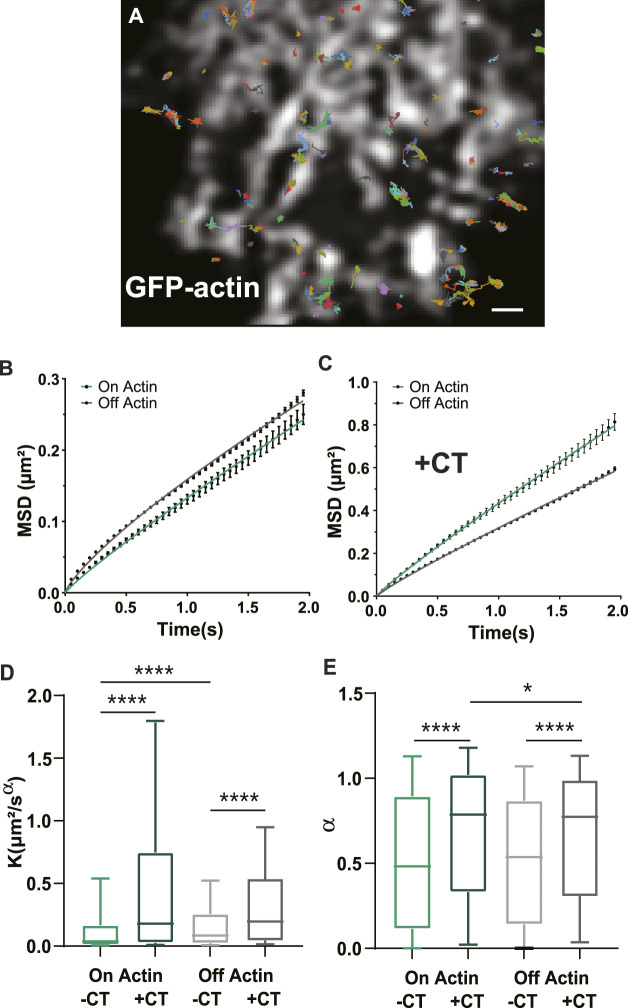
GLT-1a diffusion is altered by actin and the cytosolic GLT-1a C-terminus. **(A)** Overlay of GLT-1a diffusion tracks onto cortical actin in an astrocyte expressing GFP-actin. Individual trajectories are shown with different colors. **(B)** Mean square displacements (MSD) of diffusing GLT-1a molecules on actin (green) and off actin (grey), in normal conditions. **(C)** MSD plots with co-expression of cytosolic C-terminus. Lines shown are fits of the data to Eq. 3 (B: On K = 0.13, α = 0.90, *R* ^2^ = 0.998; **(A)** Off K = 0.16, α = 0.790, *R* ^2^ = 0.998; **(C)** On K = 0.43, α = 0.91, *R* ^2^ = 0.999; Off K = 0.32, α = 0.90, *R* ^2^ = 0.999). **(D)** Generalized diffusion coefficients (K) of GLT-1a molecules on actin (median = 0.0375, N = 3,022 trajectories) are significantly slower than GLT-1a molecules off actin (median = 0.084, N = 18,539 trajectories, *p* < 0.0001). Diffusion on actin significantly increased with co-expression of the GLT-1a CT (median = 0.18, N = 1677 trajectories, *p* < 0.0001). Expression of the GLT-1a CT also significantly increased the diffusion of GLT-1a off actin (median = 0.196, N = 11,443, *p* < 0.0001). **(E)** The anomalous exponent (*α*) is similar in trajectories on and off actin (On: median = 0.482, N = 3,022 trajectories; Off: median = 0.54, N = 18,539 trajectories; *p* = 0.9877). The anomalous exponent increases with co-expression of the cytosolic C-terminus in trajectories localized both on actin (median = 0.79, N = 1677 trajectories, *p* < 0.0001) and off actin (median = 0.75, N = 11,443, *p* < 0.0001). Box plots in D and E represent the median and interquartile range. Bars represent the 10th—90th percentile. Scale bar is 2 μm.

We next wanted to know whether the CT could alter GLT-1a diffusion dynamics, since GLT-1 mobility is important for buffering glutamate (Murphy-Royal et al., 2015; Al Awabdh et al., 2016). To ensure accurate diffusion measurements, we used single particle tracking and averaged the mean square displacements (MSDs) of the total population of GLT-1 molecules. In order to detect single GLT-1a molecules, anti-V5 antibody labeling was done at low density, therefore making it impossible to distinguish nanoclusters from single transporters. Thus, our collected trajectories of GLT-1a molecules included both freely diffusing and static molecules and nanoclusters. An example of collected trajectories is shown in Figure 4A and these single particle tracks were separated according to their location relative to actin. Mean square displacements under normal conditions show that diffusion on and off actin (green and grey lines, respectively) are different, with GLT-1a molecules off actin diffusing more than those on actin (Figure 4B, *p* < 0.0001). Expression of the CT increased the diffusion both on and off actin (Figures 4C, D). Note the different *y*-axis scales when comparing panels B and C. In the presence of the CT, the generalized diffusion coefficient K of GLT-1a molecules on actin increased by a factor of 4.8 (*p* < 0.0001), and off actin by a factor of 2.3 (*p* < 0.0001) (Figure 4D). In addition, analysis of the anomalous exponent *α*, which describes deviations from free diffusion, revealed trajectories of GLT-1a on actin filaments (a = 0.52 ± 0.01) are much more static than those measured in the presence of the CT (a = 0.69 ± 0.01, *p* < 0.0001, Figure 4E). Likewise, trajectories of GLT-1a off actin filaments (a = 0.527 ± 0.003) are more subdiffusive than those measured in the presence of the CT (a = 0.664 ± 0.004, *p* < 0.0001, Figure 4E). These data imply the C-terminus of GLT-1a is important for limiting GLT-1a diffusion both on actin and off actin.

Since previous studies of GLT-1 diffusion found that 100 µM glutamate increased diffusion (Murphy-Royal et al., 2015; Al Awabdh et al., 2016), we also examined the effect of glutamate on GLT-1 diffusion and nanoclustering. The data presented in Supplementary Figure S5 indicate a significant increase in motility in response to glutamate while also demonstrating that GLT-1a nanocluster localization on actin filaments does not change after glutamate addition. It appears the effect of glutamate on diffusion primarily affects free transporters, although the underlying mechanism therein remains elusive.

### 3.5 GLT-1a in the astrocyte membrane surrounds neuronal Kv2.1 clusters

**FIGURE 5:**
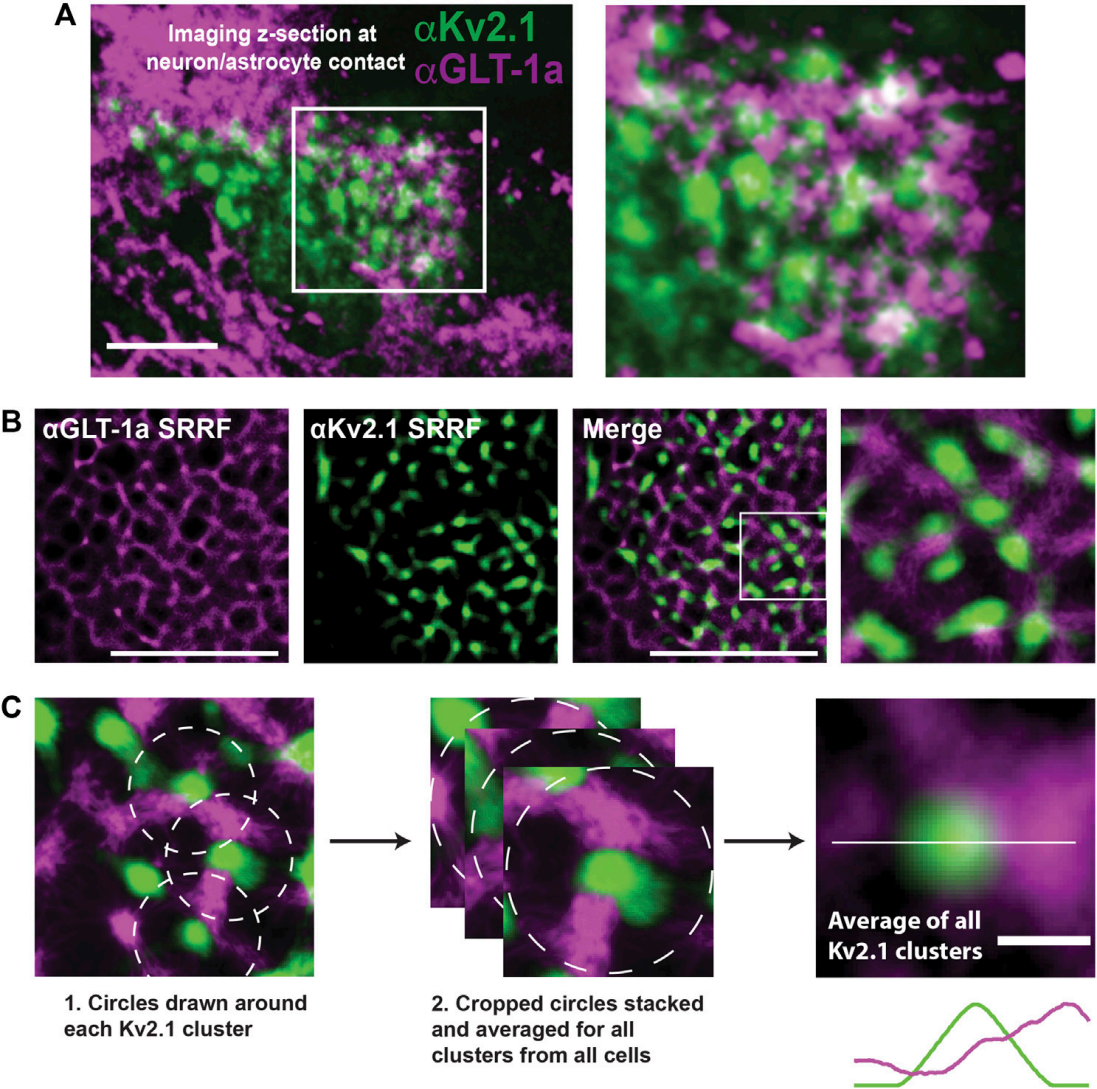
Relationship between neuronal Kv2.1 clusters and astrocytic GLT-1a in hippocampal neuron-astrocyte co-cultures. After 14 days in culture hippocampal cells were immune-labeled with antibodies specific for Kv2.1 and GLT-1a (mouse anti-Kv2.1 from NeuroMab, Davis, CA, and rabbit anti-EAAT2 from Abcam, Cambridge, MA). **(A)** Image of a pyramidal cell with a portion of the basal surface in contact with a GLT-1a positive astrocyte. The right panel shows an enlargement of the boxed area. Scale bar = 10 µm. **(B)** Representative SRRF images of the immuno-localization pattern of endogenous astrocytic GLT-1a (magenta) around endogenous Kv2.1 in neurons (green). These images represent a single z-plane between a neuron and astrocyte. Scale bars = 5 µm. **(C)** All the Kv2.1 clusters (N = 9 cell pairs, n = 495 clusters) were averaged to create the image on the right panel, showing GLT-1 surrounds Kv2.1 clusters, with occasional co-localization. Scale bar = 0.5 µm.

Thus far we have examined GLT-1a behavior in isolated DIV8 astrocytes within our hippocampal cell cultures. However, previous work indicates that astrocytic GLT-1 transporters reside in net-like structures around neuronal Kv2.1 clusters in somatosensory cortex (Misonou et al., 2008), suggesting neuronal structures may influence astrocytic GLT-1a localization. To determine whether we could replicate these data *in vitro*, hippocampal co-cultures of neurons and astrocytes were cultured for 14 days, subsequently fixed, and immune-labeled for endogenous Kv2.1 in neurons and endogenous GLT-1a in astrocytes. We moved to DIV14 since at this culture age there are well developed neuronal dendrites and axons in addition to extensive astrocytic processes (Dittmer et al., 2017; Wild et al., 2019; Panzera et al., 2022). As illustrated in Figure 5A, neuronal cell bodies can be found growing on top of astrocytes where astrocytic GLT-1a often avoids membrane directly adjacent to the neuronal surface where the Kv2.1 channels are clustered. Note that here GLT-1a nanoclusters are not obvious due to the high surface density. To best examine the spatial relationship between the neuronal Kv2.1 clusters and astrocytic GLT-1a, we again used SRRF as illustrated in Figure 5B. Focusing on the z-plane where neurons and astrocytes came into contact, SRRF also revealed a net-like localization of GLT-1 around Kv2.1 clusters. To determine the average relationship of astrocytic GLT-1a and neuronal Kv2.1, every Kv2.1 cluster and the surrounding areas were averaged as illustrated in Figure 5C. Using this analysis (N = 9 cell pairs, n = 495 clusters), we found that GLT-1a rarely overlaps with neuronal Kv2.1 (Figure 5C, third panel). A line scan through the center of the averaged image shows lower GLT-1a fluorescence (magenta) in the astrocytic membrane directly across from Kv2.1 cluster peak fluorescence (green).

We next asked whether the neuronal Kv2.1 clusters could restrict GLT-1a diffusion in the apposed astrocyte membrane, for the AMIGO beta subunit for Kv2.1 could be interacting with something in the astrocyte membrane to create a diffusion restricted space. However, the FRAP experiments presented in Supplementary Figure S6 indicate this is not the case, suggesting that the concentration of GLT-1 in nets around Kv2.1 as observed in the super-resolution images of Figure 5 is likely not due to restricted diffusion. FRAP was used here because we suspect single particle tracking with the extracellular V5 antibody is sterically hindered at the neuron-astrocyte interface.

### 3.6 Astrocytic actin colocalizes with GLT-1a around neuronal Kv2.1 clusters

**FIGURE 6:**
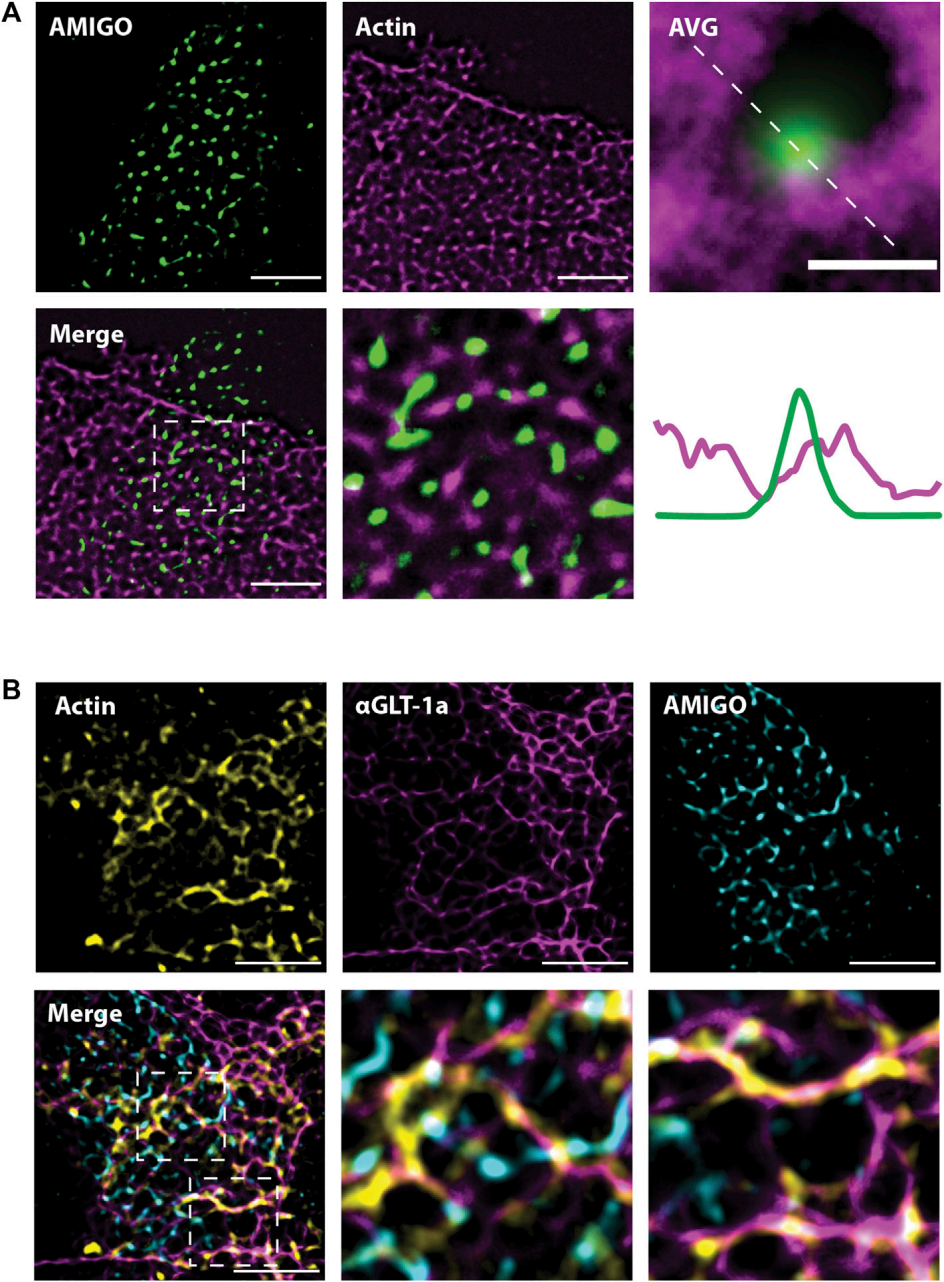
Astrocytic actin co-localizes with GLT-1 in nets around neuronal Kv2.1 clusters. Images were acquired according to the same protocol outlined in Figure 6A. Each image shown here represents a single z-plane at the junction between a neuron and an astrocyte. **(A)** Representative SRRF images of astrocytic Ruby2-actin (magenta) localizing in nets around neuronal AMIGO (green), which resides in Kv2.1 clusters. Every Kv2.1 cluster from every cell (N = 15 cell pairs, n = 6,666 Kv2.1/AMIGO clusters) was averaged to create the image on the right, showing astrocytic actin surrounds Kv2.1 clusters, with rare co-localization. Scale bar = 0.5 µm. **(B)** Representative images showing astrocytic Ruby2-actin (yellow) and GLT-1 (magenta) co-localize and together form nets around neuronal Kv2.1/AMIGO clusters (cyan). N = 10 cell pairs. Scale bars = 5 µm.

The experiments illustrated in Figures 2, 3 suggest actin is involved in the localization of astrocytic GLT-1. Thus, we next wanted to know whether the location of astrocytic actin could be influenced by the presence of Kv2.1 clusters on the adjacent neuronal membrane. To this end, we expressed Ruby2-Actin specifically in astrocytes using the gfaABC1D promoter. Simultaneously, we expressed a GFP-tagged AMIGO1 specifically in neurons using the SYN promoter to visualize the Kv2.1 clusters. The use of cell-specific promoters was essential to ensure the imaged actin was actually located in astrocytes. Using SRRF and focusing on the plane where the 2 cell types had the most contact, we found that astrocytic actin also displayed a net-like localization pattern around neuronal Kv2.1 clusters (Figure 6A). Indeed, when all the clusters were averaged as in Figure 5, astrocytic actin mostly occupied the region directly around Kv2.1 clusters (Figure 6A, lower right panel). Finally, using a combination of Ruby2-actin expression in astrocytes with GFP-AMIGO1 expression in neurons and GLT-1a immune-labeling we discovered that astrocytic actin and GLT-1 co-localize in the net surrounding neuronal Kv2.1 clusters (Figure 6B), suggesting the GLT-1a-actin relationship is responsible for the localization pattern observed previously (Misonou et al., 2008). Altogether, these data suggest the GLT-1a-actin interaction is necessary for localization near neuronal structures involved in glutamate sensing.

## 4 Discussion

In order to maintain the fidelity of glutamate neurotransmission, astrocytes use highly expressed glutamate transporters, such as GLT-1, to limit glutamate signals spatially and temporally (Weng et al., 2007; Pinky et al., 2018). Here, we describe the localization of GLT-1a nanoclusters in relation to cortical actin filaments using a combination of super-resolution microscopy and single particle tracking. Our results indicate that GLT-1a and GLT-1b can both form nanoclusters in astrocytes and HEK cells and both isoforms can occupy the same nanoclusters. However, these isoforms differed in their localization, such that GLT-1a nanoclusters strongly associated with cortical actin filaments, while GLT-1b nanoclusters did not. Both GLT-1a and GLT-1b nanoclusters had higher fluorescence intensity when localized on actin filaments, indicating an increased number of transporters per nanocluster. Expression of a cytosolic GLT-1a C-terminus protein disrupted the localization and fluorescence intensity of GLT-1a nanoclusters on actin filaments, suggesting the C-terminus interacts with cellular components required for actin association. Expression of the C-terminus also increased overall diffusion of GLT-1a transporters both on and off actin filaments, indicating the C-terminus likely binds proteins playing a role in governing transporter diffusion. Nanocluster localization on actin was undisturbed by glutamate application, suggesting that glutamate binding and transport does not strongly affect localization of GLT-1 nanoclusters. Finally, using astrocyte-neuron co-cultures we showed that the actin-GLT-1a interaction is important in localizing GLT-1 transporters adjacent to key neuronal structures, as astrocytic actin and endogenously expressed GLT-1 co-localized in nets around neuronal Kv2.1 clusters, which are themselves regulated by high extracellular glutamate.

### 4.1 Nanoclustering is a conserved feature of both GLT-1 splice forms

The data presented here suggest that two isoforms of GLT-1, GLT-1a and GLT-1b, are capable of forming nanoclusters (Figure 1), which implies a shared mechanism initiates nanocluster formation, possibly via interaction with membrane cholesterol as suggested previously (Butchbach et al., 2004; Sullivan et al., 2004; Raunser et al., 2006). Here, we counted the number of V5 antibodies bound per nanocluster as a proxy for the number of transporters localized in this compartment (Figures 1B, D), for it is difficult to know the number of antibodies that could bind to an individual transporter due to potential steric hindrance. However, due to the fact that GLT-1a and GLT-1b are identical except for the C-terminal sequence, it is unlikely the number of antibodies bound per transporter would vary between these two splice variants. Therefore, differences between GLT-1a and GLT-1b nanoclusters are likely due to distinct localization mechanisms. We also found that only GLT-1a nanoclusters are localized near actin, indicating there is something about this cytoskeletal environment that both localizes GLT-1a nanoclusters (Figures 2A, C) and increases the number of GLT-1a transporters per nanocluster (Figure 2E). Together with Figure 1, these results indicate multiple mechanisms can regulate GLT-1 nanocluster formation and location.

### 4.2 Cortical actin is central to GLT-1a nanocluster location

Our understanding of plasma membrane organization and architecture has evolved from the Singer-Nicholson fluid mosaic model, which postulated a homogeneous lipid bilayer embedded with freely diffusing proteins (Singer and Nicolson, 1972). The current prevailing model is far more complex, with the plasma membrane consisting of heterogeneous patches of lipids and proteins, which are dynamically regulated (Maxfield, 2002; Kusumi et al., 2011; Nicolson, 2013; Garcia-Parajo et al., 2014; Goñi, 2014; Krapf, 2015). Compartmentalization of the plasma membrane is thought to improve regulation efficiency and provide specialized signaling domains (Garcia-Parajo et al., 2014; Krapf, 2015). Protein constituents of these domains are manipulated by lipid composition and turnover, extracellular matrix contacts, and cytoskeletal encounters (Kalappurakkal et al., 2020).

A fine mesh of cortical cytoskeleton filaments that lie just beneath the plasma membrane acts as a diffusion barrier (Köster and Mayor, 2016; Sadegh et al., 2017) and nanocluster nucleator (Gowrishankar et al., 2012; Sil et al., 2020). Cortical cytoskeleton filaments, composed of actin and septins, can simultaneously limit the lateral diffusion of membrane proteins and facilitate interactions between membrane proteins or lipids and cytoskeletal components to generate nanoclusters (Kalappurakkal et al., 2020). The work in this paper suggests this mechanism is relevant to glutamate transporters as well. However, GLT-1a nanoclusters clearly do not directly bind cortical actin, for nanoclusters were often found adjacent to actin as opposed to being truly colocalized when using the SRRF super-resolution approach. Therefore, it is possible that the GLT-1a C-terminus interacts with unknown proteins distally associated with the cortical actin cytoskeleton.

While the data presented here focus on localization near the actin cytoskeleton, the septin cytoskeleton is intimately connected and dependent upon the actin cytoskeleton (for review: (Spiliotis, 2018). We found GLT-1a was localized on and adjacent to actin filaments (Figure 2), perhaps implicating the septin cytoskeleton in GLT-1a localization. Septins co-localize prominently with certain features of actin filaments (Spiliotis, 2018), most notably stress fibers and focal adhesions, which are necessary to maintain peripheral astrocyte processes and stabilize cell adhesions (Guasch et al., 2003). Multiple glutamate transporters, including GLT-1, are localized to the tips of such processes (Hayashi and Yasui, 2015), which might rely on a septin interaction. However, actin depolymerization (Supplementary Figure S4) did not alter GLT-1a nanocluster density or number so perhaps a septin network remains intact after actin filament removal. Additional experiments will be required to elucidate the actin/GLT-1a nanocluster link.

### 4.3 Nanoclustering may impact transporter function

Nanoclustering is thought to be important in regulating several features of membrane protein function, such as concentrating ligand binding sites, improving signal transduction, and allowing allosteric cooperation (Garcia-Parajo et al., 2014). Certainly, concentration of GLT-1 transporters near synapses is vital in limiting glutamate neurotransmission. Interestingly, neuronal activity induced by gabazine increased GLT-1 nanocluster diameter by 49% and decreased GLT-1 nanocluster distance to synapses (Benediktsson et al., 2012). Although we did not observe increases in GLT-1a nanocluster fluorescence intensity after glutamate addition, which should be comparable to nanocluster diameter, it is difficult to equate the neuronal activity elicited by gabazine and the concentration of glutamate used in this study. Furthermore, GLT-1 transporters are rapidly internalized under conditions of high extracellular glutamate, such as in ischemia and traumatic brain injury (Ibáñez et al., 2016). Given the potential importance of actin in endocytosis (Francis et al., 2023; Wu and Wu, 2023; Yu and Yoshimura, 2023), localizing GLT-1 nanoclusters near actin filaments could be a mechanism to swiftly internalize glutamate-bound transporters unable to function due to ionic gradient perturbations in pathophysiological conditions. Additionally, nanocluster perturbation via cholesterol depletion decreased transport efficiency by ∼30%, suggesting that nanoclustering of GLT-1 may be functionally relevant for transporter function (Raunser et al., 2006). In another study, cholesterol disruption resulted in rapid internalization of GLT-1 transporters, suggesting that GLT-1 surface stability may be related to transporter function (Butchbach et al., 2004). Whether nanoclustering affects transport efficiency directly or by increasing GLT-1 transporter stability in the membrane awaits future investigation.

### 4.4 Looking towards the GLT-1 interactome

Biochemical approaches have identified several proteins that could act as GLT-1 interactors, including cytoskeleton-associated proteins Ajuba (Marie et al., 2002) and Sept2-associated BORG4 (Piniella et al., 2018), PDZ proteins PICK1 (Bassan et al., 2008; Zou et al., 2011) and MAGI1 (Zou et al., 2011), the Na^+^/K^+^ ATPase α-subunit (Genda et al., 2011), and various mitochondrial proteins (Genda et al., 2011). Any one of these interactors could reasonably contribute to immobilization of GLT-1 molecules on the astrocytic surface, both near neuronal synapses and somatic Kv2.1 clusters. The present work suggests the primary mechanism of GLT-1a nanocluster immobilization is via an interaction with an actin-associated protein (Figure 2, Figure 4B). An Ajuba interaction is unlikely to explain the observations presented here because the N-terminus of GLT-1 is thought to regulate the interaction, which is identical between GLT-1a and GLT-1b. Also, our results in Figures 3, 4 implicate the GLT-1a C-terminus in regulating the localization of nanoclusters to cortical actin filaments. The specific amino acid residues involved in the BORG4/Sept2 interaction with GLT-1 have not yet been identified, and this partnership should be the focus of future investigations.

Interestingly, GLAST, another highly expressed glutamate transporter, interacts with Sept2 in Bergmann glia via the GLAST C-terminus (Kinoshita et al., 2004). More recent evidence suggests that this interaction is dependent on the septin effector, BORG4, and is crucial in localizing GLAST to perisynaptic astrocyte membranes (Ageta-Ishihara et al., 2015). Mislocalization of GLAST caused impairment in the time course of glutamate clearance, suggesting the perisynaptic localization of GLAST is imperative for proper glutamate signaling dynamics in the cerebellum. Together with the data presented in this work, this suggests cytoskeletal interactions may be a ubiquitous method of localizing glutamate transporters.

### 4.5 Neuron-astrocyte adhesions

Neuron-astrocyte adhesions occur at both tripartite synapses (Hillen et al., 2018) and clusters of Kv2.1 channels on the neuronal soma (Du et al., 1998). The mechanism of GLT-1 localization near these neuronal structures is unknown. However, considering the localization of GLT-1 and the heavy involvement of actin in cell-cell contact (Yamazaki et al., 2007; Tang and Brieher, 2013), the GLT-1a-actin interaction is likely involved in GLT-1 localization near both of these neuron-astrocyte adhesions. Indeed, in the present study, we found that both astrocytic GLT-1 and actin filaments were localized in nets around Kv2.1 clusters in neurons (Figures 5, 6), suggesting the GLT-1-actin relationship is important for this pattern of localization near neuronal Kv2.1 clusters.

**FIGURE 7:**
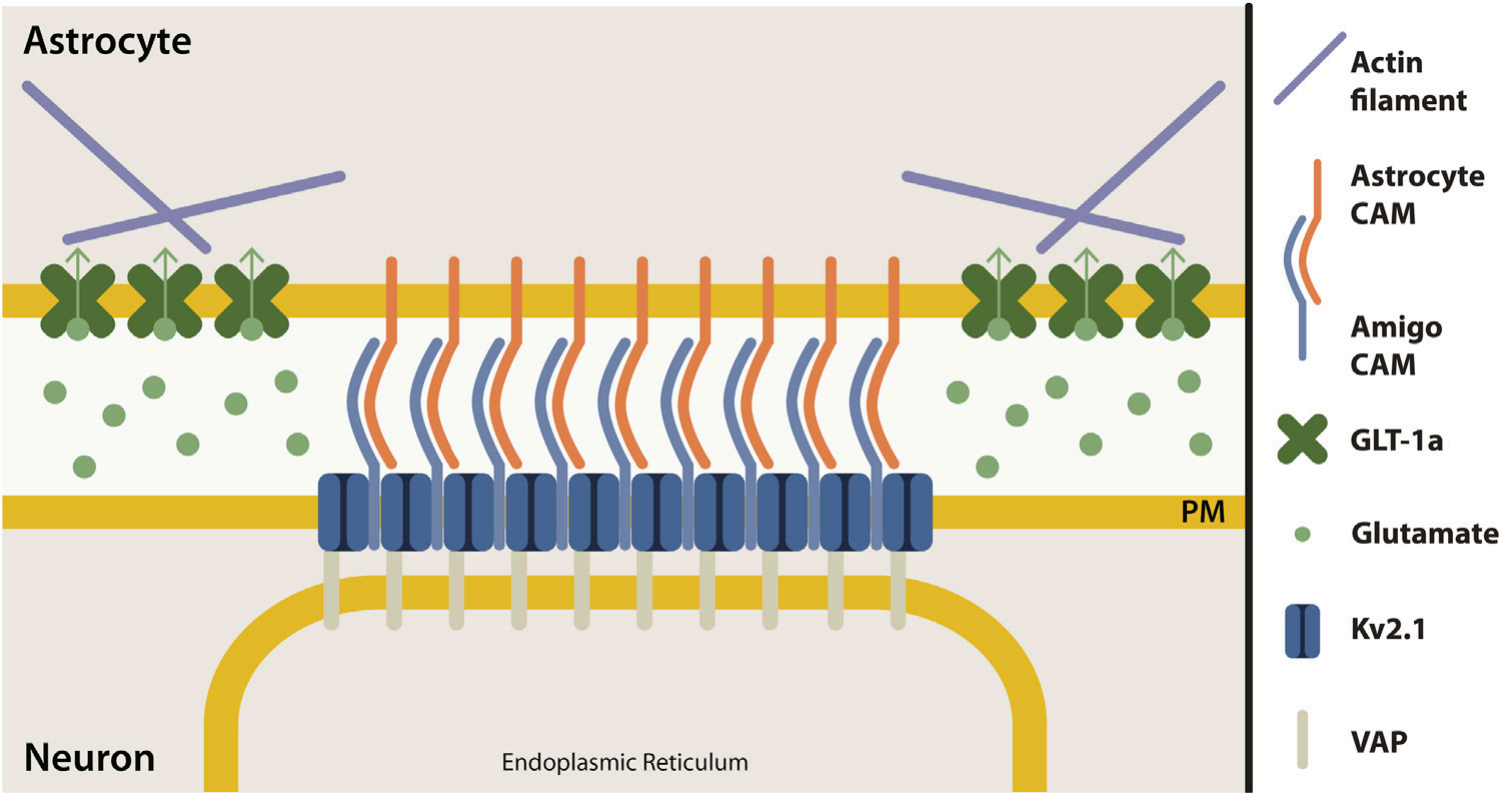
Proposed model of neuron-astrocyte contact site at Kv2.1 cluster. Clusters of Kv2.1 channels (dark blue) in the neuronal plasma membrane (yellow) are sites where neurons and astrocytes make contact. Kv2.1 channels have an auxiliary subunit, AMIGO (light blue), which is a cell adhesion molecule (CAM). Presumably, an astrocyte cell adhesion molecule (orange) interacts with AMIGO to bring neurons and astrocytes together at this junction. Astrocyte GLT-1 transporters (green) are localized in nets around neuronal Kv2.1 clusters, perhaps due to the interaction of GLT-1a with actin (purple). Kv2.1 clusters also form ER-PM junctions by interacting with the ER protein, VAP (grey).

The micron-sized clusters of Kv2.1 channels on the somatic membrane of central neurons represent sites of endoplasmic reticulum/plasma membrane junctions (Du et al., 1998; Fox et al., 2015; Johnson et al., 2018; Kirmiz et al., 2018). These Kv2.1 clusters are localized adjacent to both astrocyte and microglia processes in the murine brain (Du et al., 1998; Cserép et al., 2020), adhesion sites which might be regulated or formed by the Kv2.1 auxiliary subunit, AMIGO, a cell adhesion molecule (Kuja-Panula et al., 2003; Peltola et al., 2011). Although the adhesion molecules involved in Kv2.1-astrocyte contact are currently unknown, it seems that, like junctions in endothelial cells, this adhesion site is capable of regulating actin filaments. Our working model of this adhesion site is depicted in Figure 7; however, the mechanism by which somatic Kv2.1 clusters regulate astrocytic actin is an open question.

### 4.6 The neuron-astrocyte junction and the response to ischemic insult

The localization of astrocytic GLT-1 in nets around neuronal Kv2.1 clusters ((Misonou et al., 2008) and Figure 5B)) likely has a homeostatic role that can be overwhelmed under pathophysiological conditions. Ischemia, excitotoxicity, or pharmacological inhibition of GLT-1 function cause a rapid dispersal of clustered Kv2.1 channels, leading to cortical ER retraction within the neuron (Misonou et al., 2008; Mulholland et al., 2008; Fox et al., 2015) and likely altered neuronal Ca^2+^ homeostasis (Vierra et al., 2019; Panzera et al., 2022; Vierra et al., 2023). Following ischemic insult, reduced astrocytic glutamate uptake activates extrasynaptic NMDA receptors (Mulholland et al., 2008), where the resulting Ca^2+^ influx induces calcineurin-dependent dephosphorylation within the Kv2.1 C-terminus. Channel dephosphorylation breaks contact with the ER VAPs. Perhaps actin-based concentration of astrocytic GLT-1a nanoclusters adjacent to the neuronal Kv2.1 microclusters exists to ensure Kv2.1-mediated ER/PM junctions remain under normal levels of extracellular glutamate. Only when these transporters are inhibited following ischemic insult are the Kv2.1-induced ER/PM junctions lost in the adjacent neuron. While NMDA receptors do not truly colocalize with somatic Kv2.1 clusters (Mulholland et al., 2008), it is likely that adjacent glutamate receptors on the soma regulate Ca^2+^ influx which impacts the Kv2.1 interaction with the ER to promote Kv2.1 declustering, as described above (Misonou et al., 2008). In addition, electron microscopy studies of membrane contact sites *in vivo* indicate excitatory synapses are often near somatic ER/PM junctions (Wu et al., 2017), thus excitatory input onto the soma could also regulate Kv2.1 ER/PM interaction. Whether astrocytic GLT-1a localization is altered following declustering of the Kv2.1/AMIGO adhesion molecule complex is an area for future study. Altogether, these studies suggest the Kv2.1-astrocyte contact is an important sensor for neuronal insult. In addition, Kv2.1/microglia contacts appear to play a neuroprotective role following experimental stroke (Cserép et al., 2020).

## 5 Conclusion

These data indicate that the GLT-1a-actin relationship may be important in determining the localization of GLT-1a near neuronal ER/PM junctions that are sensitive to excess glutamate. Due to the slow transport cycle of GLT-1a, it is imperative that transporters are localized at the right place at the right time. Understanding the mechanisms regulating GLT-1a C-terminal interaction with the cytoskeleton, and thus transporter localization, will be essential to identify new targets for mitigation of neuronal insults which lead to high ambient glutamate, such as ischemic stroke, traumatic brain injury, and epilepsy.

## Data Availability

The raw data supporting the conclusion of this article will be made available by the authors, without undue reservation.
