# Supplementary material for: GLT-1a glutamate transporter nanocluster localization is associated with astrocytic actin and neuronal Kv2 clusters at sites of neuron-astrocyte contact
Source: Front Cell Dev Biol. 2024 Feb 1;12:1334861. doi: 10.3389/fcell.2024.1334861 (PMC10867268; doi:10.3389/fcell.2024.1334861)
Supplement: Supplementary file 1 [file DataSheet1.docx]

Supplementary Material

# 1. Supplementary Methods

**Fluorescence recovery after photobleaching (FRAP).** Astrocytes expressing gfaABC1D>Ruby2-GLT-1a-V5 in contact with neurons expressing AAV9:SYN>AMIGO-GFP were imaged simultaneously using spinning disk confocal microscopy. Movies were acquired at 1 Hz for 500 s at the plane where the two cells came into the most contact. The first ten frames were collected to measure initial fluorescence. We then bleached the astrocyte surface with a high intensity 561 nm laser, which ensured only the GLT-1a was photobleached. Photobleaching resulted in approximately 80% loss of fluorescence after background subtraction. A mask was created from the AMIGO-GFP signal and subsequently used to measure the recovery of GLT-1a diffusion inside and outside of this region. Fluorescence measurements in each region were normalized to the average of the first 10 frames.

#
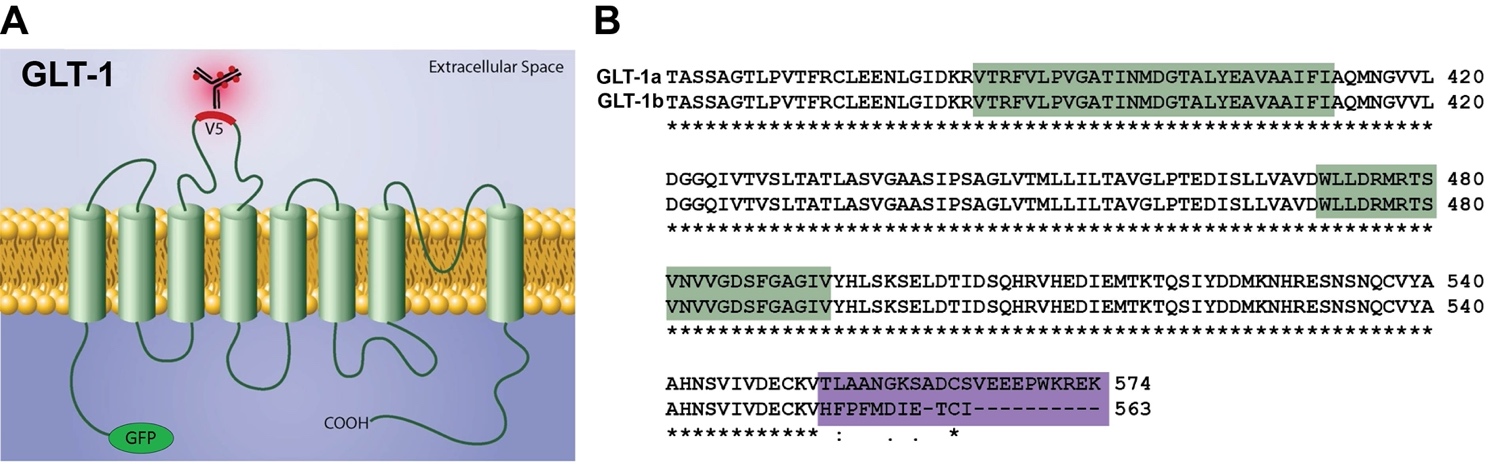
2. Supplementary Figures

## SUPPLEMENTARY FIGURE S1

# Summary of the GLT-1 constructs used in this study. (A) Location of the V5 epitope tag and fluorescent protein addition to both GLT-1a and 1b. (B) C-terminal amino acid sequence comparison between GLT-1a and GLT-1b.

##
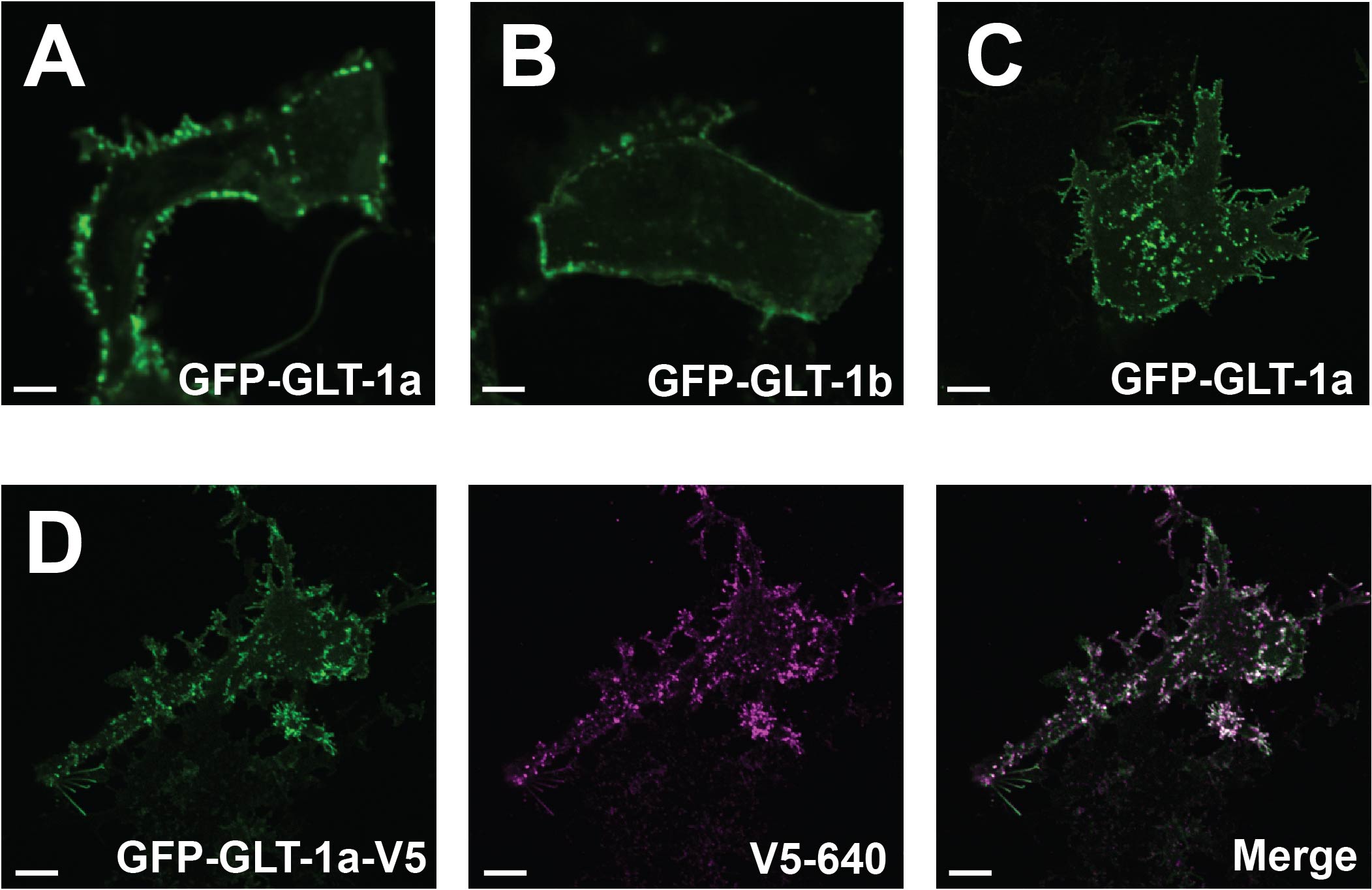


## SUPPLEMENTARY FIGURE S2

## Majority of transfected Glt1 localized to the plasma membrane in live cultured astrocytes. Most transfected GFP-GLT-1a (A) and GFP-GLT-1b (B) is localized to the surface of a cultured astrocyte, as seen in these optical confocal sections through the center of each cell. (C) The basal surface of an astrocyte expressing GFP-GLT-1a shows nanocluster localization to filopodial process tips. (D) An astrocyte expressing GFP-GLT-1a-V5, which has been labeled with an antibody directed against V5 and conjugated to a CF640 molecule, also shows localization to filopodia process tips and nanoclusters on the basal surface. Notably, there do not appear to be more or larger nanoclusters in the live cell labeled with the V5 antibody. Scale bars are 10 μm. Each image depicts a single z-plane.

##
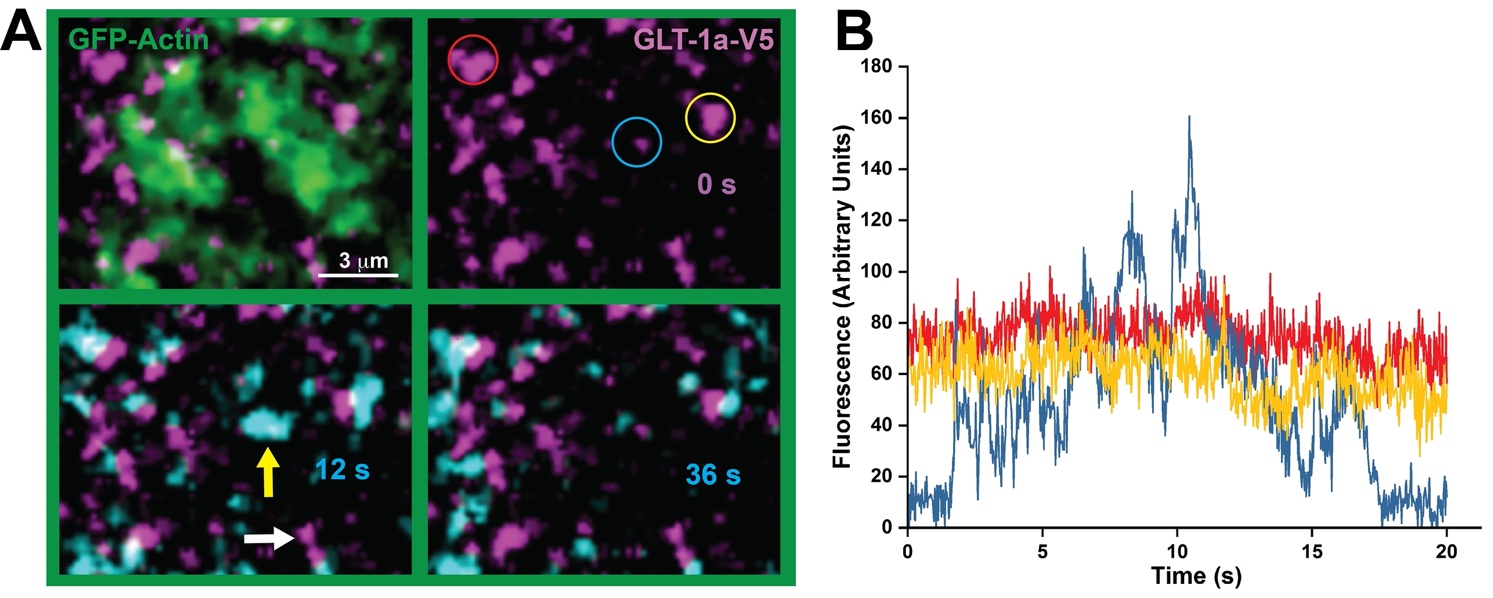


## SUPPLEMENTARY FIGURE S3

## GLT-1a nanocluster stability is highly variable. Live DIV 14 hippocampal cultures expressing Ruby2-GLT-1a-V5 and GFP-actin were labeled with CF640-conjugated anti-V5 antibody and time-lapse imaging performed. (A) Top left panel illustrates the relationship between actin and surface GLT-1a as detected with the anti V5-antibody. The remaining panels show the location of GLT-1a nanoclusters at the indicated time points, with the GLT-1a labeling at the 0 s timepoint represented in magenta in all panels while nanoclusters at the 12 and 36 s time points are shown in cyan. Use of two colors for the different timepoints illustrates nanocluster stability and dynamics. The yellow arrow points to a nanocluster that transiently appears (blue trace in B) while the white arrow indicates a nanocluster that disappears, as indicated by the lack of cyan at the 12 and 36 s timepoints. (B) Anti-V5 antibody fluorescence intensity as a function of time within the three ROIs indicated in the top right panel of A. Note that two nanoclusters are stable over the 20 s while the third transiently appears.

##
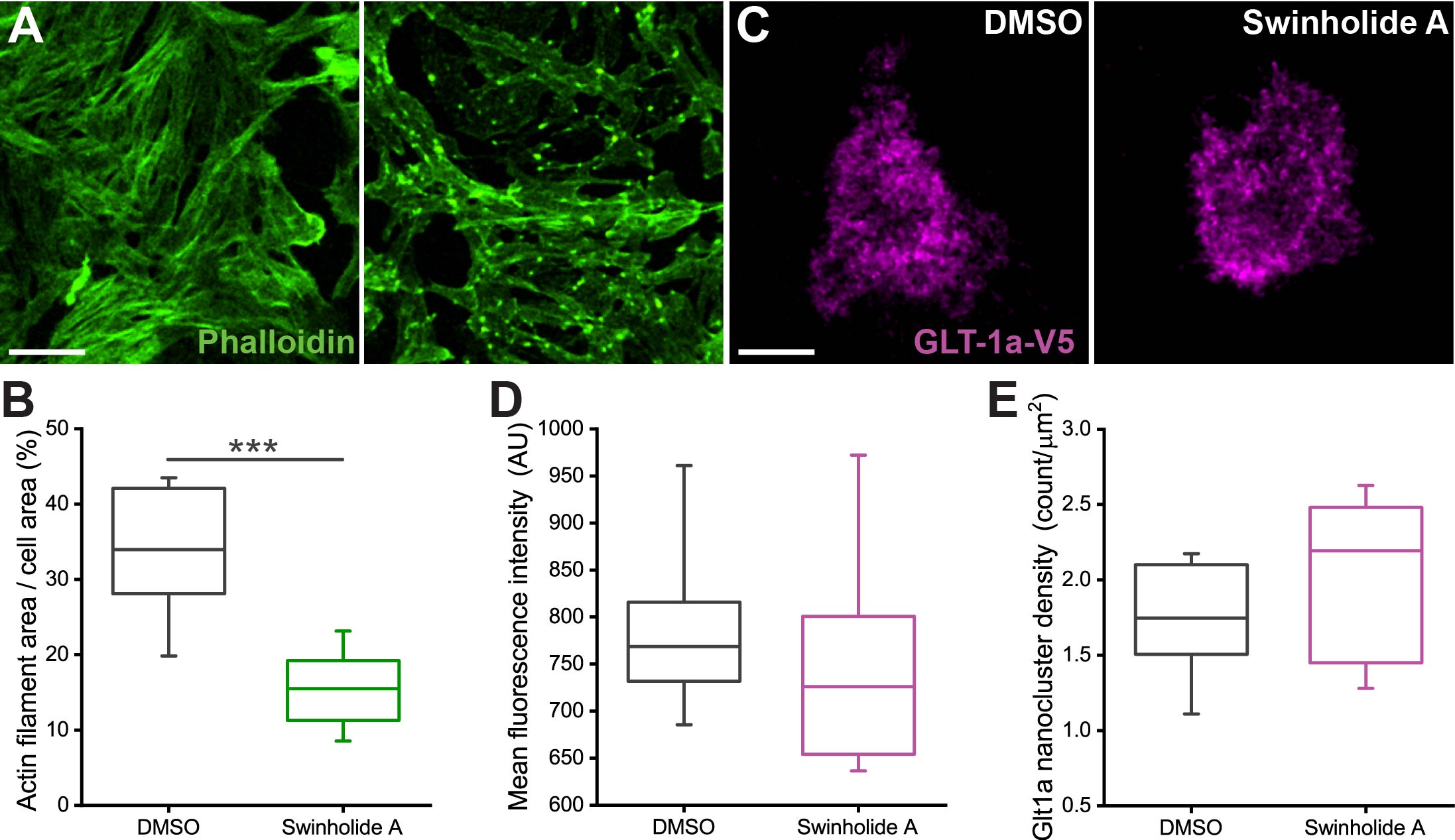


## SUPPLEMENTARY FIGURE S4

## Swinholide A-induced actin depolymerization has no effect on astrocytic GLT-1a nanoclusters. (A) Images from a DIV7 astrocyte culture treated with vehicle (0.1% DMSO, left) or 200 nM swinholide A (right) for 6 hours in maintenance media. Cells were fixed in PHEM buffer (60 mM PIPES, 25 mM HEPES, 5 mM EGTA, 1 mM MgSO_4_, pH 7.0) with 0.1% Triton X-100, 3.7% paraformaldehyde, and 0.25% glutaraldehyde and subsequently stained with Alexa-Fluor 568-conjugated phalloidin. Note the actin aggregation in treated cells. Scale bar is 100 μm. (B) Swinholide A application resulted in actin depolymerization and a reduction in the percent of total cell area occupied by actin filaments. (N=10 images, p<0.0001). (C) DIV9 astrocytes expressing GLT-1a-V5 were treated with vehicle (left) or 200 nM swinholide A (right) for 5 hours and labeled with a CF640R-conjugated anti-V5 antibody. Cells were imaged live. Scale bar is 10 μm. (D) Fluorescence intensity of GLT-1a-V5 nanoclusters did not change with swinholide A treatment (N=14 DMSO cells, N=22 swinholide A cells, p=0.745). (E) GLT-1a-V5 nanocluster count proportional to cell area did not change with swinholide A treatment (N=14 DMSO cells, N=22 swinholide A cells, p=0.128). All fluorescence measurements in arbitrary units (AU). Box plots represent the median and interquartile range. Bars represent the 10^th^ – 90^th^ percentile.

**
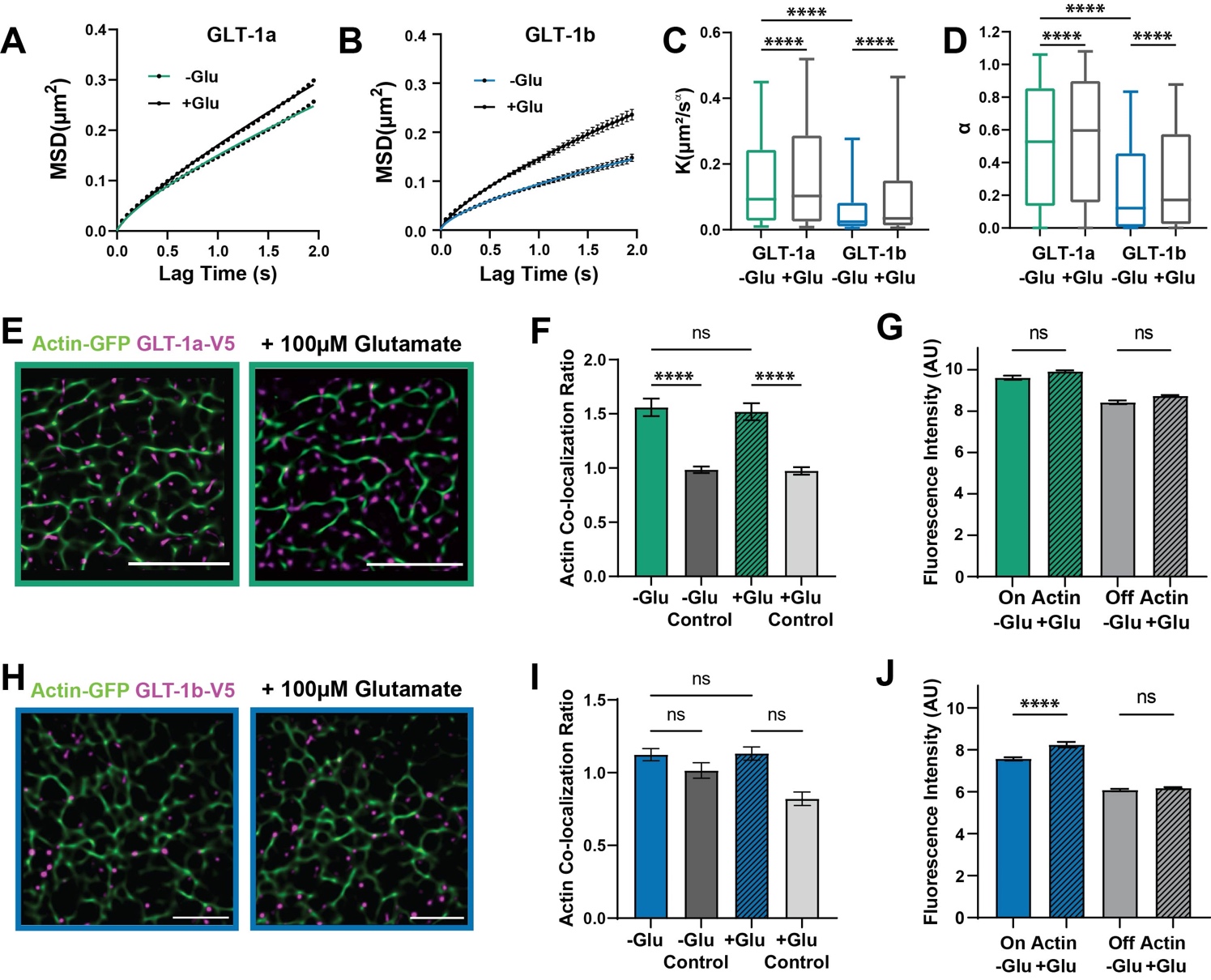
**

## SUPPLEMENTARY FIGURE S5

## Glutamate alters GLT1 diffusion but does not affect nanoclustering. Mean square displacement (MSD) as a function of lag time (s) shows an increase in diffusion of GLT-1a (A) and GLT-1b (B) 3 min after 100 µM glutamate application. Lines shown are fits of the data to Eq. 3 (A: K=0.15, α=0.75, R^2^=0.998; A: +Glu K=0.17, α=0.80, R^2^=0.998; B: K=0.09, α=0.64, R^2^=0.999; B: +Glu K=0.15 α=0.71, R^2^=0.999). (C) The anomalous diffusion coefficient (K) of GLT-1a (green, median=0.093, N=32432 trajectories) or GLT-1b (blue, median=0.025, N=2821 trajectories) significantly increases with glutamate application (GLT-1a +Glu: median=0.103, N=34540 trajectories, p<0.0001; GLT-1b +Glu: median=0.035, N=3133 trajectories, p<0.0001). The generalized diffusion coefficient for GLT-1a was significantly higher than GLT-1b (p<0.0001). (D) The anomalous exponent (α) of GLT-1a (green, median=0.53, N=32432 trajectories) or GLT-1b (blue, median=0.121, N=2821 trajectories) significantly increases with glutamate application (GLT-1a +Glu: median=0.60, N=34540 trajectories, p<0.0001; GLT-1b +Glu: median=0.171, N=3133 trajectories, p<0.0001). The anomalous exponent for GLT-1a was significantly higher than GLT-1b (p<0.0001). (E) Representative SRRF images of GLT-1a nanoclusters (magenta) overlying actin (green) before and after addition of 100 µM glutamate. (F) GLT-1a nanoclusters are concentrated to actin filaments even after addition of 100 µM glutamate (mean=1.52±0.079, N=58 cells) versus a random pixel control (0.97±0.034, N=58 cells, p<0.0001). (G) GLT-1a nanocluster sum intensity on actin filaments (9.61±0.0001, N=43 cells, 34774 nanoclusters) does not change after addition of 100 µM glutamate (9.91±0.0058, N=27 cells, 34615 nanoclusters, p=0.239). GLT-1a nanoclusters localized off actin (8.42±0.008, N=43 cells, 219464 nanoclusters) also do not change in intensity after glutamate application (8.73±0.004, N=43 cells, 204549 nanoclusters, p=0.218) (H) Representative images of GLT-1b nanoclusters (magenta) overlying actin (green) before and after addition of 100 µM glutamate. (I) GLT-1b nanoclusters are not concentrated to actin filaments even after addition of 100 µM glutamate (1.13±0.045, N=17 cells) versus a random pixel control (0.82±0.046, N=17 cells, p<0.0001). (J) GLT-1b nanocluster sum intensity on actin filaments increases after addition of 100 µM glutamate (8.14x10^5^±9.73x10^3^, 17 cells, N=13589 nanoclusters, p<0.0001). However, GLT-1b nanoclusters localized off actin (6.13±0.004, N=17 cells, 99045 nanoclusters) do not change in intensity after glutamate application (6.18±0.004, N=17 cells, 105676 nanoclusters, p=0.986). Scale bars are 5 µm. Box plots represent the median and interquartile range. Bars in box plots represent the 10^th^ – 90^th^ percentile.


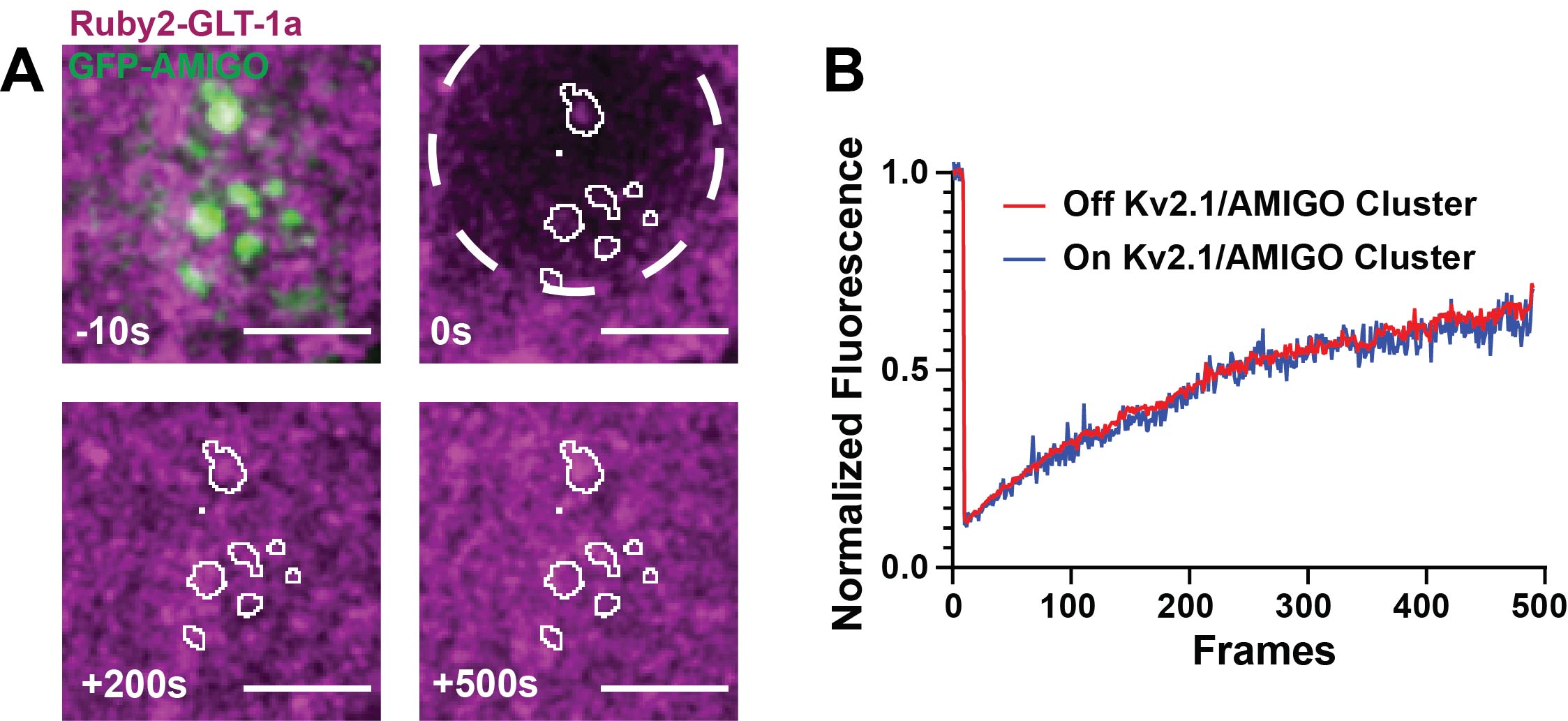


SUPPLEMENTARY FIGURE S6

## FRAP shows no differences in astrocytic GLT-1a diffusion on or off neuronal Kv2.1 clusters. (A) Representative images of Ruby2-GLT-1a (magenta) fluorescence recovery after photobleaching (FRAP). The Ruby2-GLT-1a signal is underneath a population of neuronal Kv2.1 clusters (green), labeled with the auxiliary subunit GFP-AMIGO. Images show GLT-1a fluorescence at different timepoints in a single 500 s movie. Scale bars=5 µm. (B) Normalized fluorescence shows no differences in fluorescence recovery of GLT-1a molecules at Kv2.1 clusters versus off Kv2.1 clusters in average recoveries from 10 cell pairs. Time constants (t_1/2_) were not noticeably different (On: t_1/2_=239±4 s, Off: t_1/2_=242±7 s).
